# Photodegradation of Nitrogenous Disinfection Byproducts by Far-UVC Light at 222 nm

**DOI:** 10.1021/acsestwater.5c00156

**Published:** 2025-04-10

**Authors:** Juhee Kim, Xiaoyue Xin, Ryan J. Kann, Jiaqi Li, Aidan S. Labrozzi, Jiale Xu, Ching-Hua Huang

**Affiliations:** a School of Civil and Environmental Engineering, Georgia Institute of Technology, Atlanta, Georgia 30332, United States; b Department of Civil, Environmental and Construction Engineering, University of Hawaìi at Ma̅noa, Honolulu, Hawaii 96822, United States; c School of Biology, Georgia Institute of Technology, Atlanta, Georgia 30332, United States; d Department of Civil, Construction and Environmental Engineering, North Dakota State University, Fargo, North Dakota 58102, United States

**Keywords:** disinfection byproducts (DBPs), KrCl* excimer lamp, far-UVC, water treatment, haloacetonitriles, haloacetamides

## Abstract

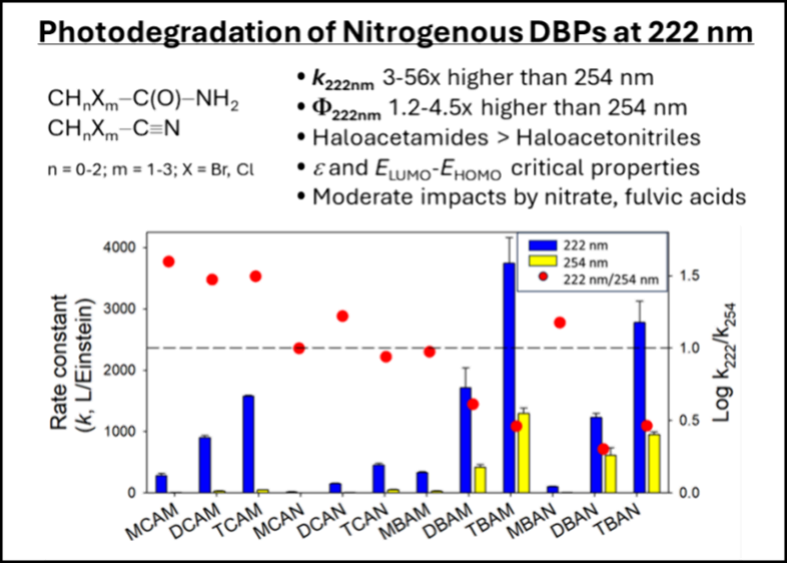

Krypton chloride (KrCl*) excimer lamps emitting far-UVC 222 nm light have emerged as a promising alternative technology to conventional low-pressure UV (LPUV) lamps emitting at 254 nm. Herein, the suitability of 222 nm for the photodegradation of 12 haloacetonitrile and haloacetamide disinfection byproducts (DBPs) was investigated. Photolysis of all these nitrogenous DBPs is significantly enhanced at 222 nm, compared to 254 nm. The photolysis rate constants (*k*_222 nm_ = 7.96 × 10^3^ – 1.60 × 10^6^ cm^2^·Einstein^–1^) and quantum yields (Φ_222 nm_ = 0.049–14.43) are 3–56 and 1.2–4.5 times greater, respectively. The photolysis rate of haloacetamides is faster than that of haloacetonitriles and increases with the number of halogens on DBPs. Bromo-DBPs feature much faster photodegradation than chloro-DBPs within the same structural class. The photolysis rates at 222 nm strongly correlate with DBP molar absorption coefficients (ε) and the energy gap between the highest occupied and the lowest unoccupied orbitals (*E*_HOMO_ – *E*_LUMO_), indicating the importance of light absorption and photoexcitation. Nitrate and natural organic matter in water cast considerable light-screening effects but also generate reactive species that play a role in the degradation of DBPs at 222 nm. These findings are useful for further developing far-UVC-based technology to mitigate water contamination.

## Abstract 2

The KrCl* excimer lamp emitting at 222 nm is a promising UV technology to enhance the photodegradation of nitrogenous DBPs, which is highly influenced by the molar absorptivity and electronic properties of DBPs.

## 1 Introduction

Ultraviolet (UV) irradiation is a promising water treatment technology owing to several advantages, such as easy maintenance, the absence of harmful residues, and elimination of additional chemical inputs.^1−3^ UV irradiation can be categorized into three main types based on wavelength: UVA (320–400 nm), UVB (280–320 nm), and UVC (200–280 nm).^4,5^ Traditional UVC irradiation at 254 nm using low-pressure mercury lamps (LPUV) has been extensively employed for disinfection. However, mercury lamps come with certain limitations, including health risks linked to direct UV exposure, mercury content, a long warm-up time, and fluctuations in radiation intensity influenced by environmental factors like temperature.^4−6^ Moreover, due to the implementation of the Minamata Convention by 128 countries,^7^ the use of mercury lamps needs to be reduced; as a result, other lamps, such as UVC light-emitting diode (LED) and excimer lamps, are expected to play a more significant role in the future UV disinfection market.^5^

The krypton chloride (KrCl*) excimer lamps emitting primarily at 222 nm are increasingly popular due to their perceived safety, owing to limited ability to penetrate human tissues and eyes at such wavelength.^8−11^ KrCl* excimer lamps also offer several other benefits, including instantaneous on–off functionality, a prolonged lifespan, and low impact by temperature.^12,13^ With advantages over conventional LPUV lamps, KrCl* excimer lamps have emerged as a promising technology for water and air disinfection.^14,15^ In recent years, interests have also grown in utilizing KrCl* excimer lamps to photodegrade organic contaminants.^16,17^ Research has shown that conventional LPUV systems are not efficient in photodegrading most organic compounds.^17,18^ In comparison, the lower wavelength of KrCl* excimer lamps provides higher photon energy for excitation of molecules (photon energy = 539 kJ·Einstein^–1^ and 471 kJ·Einstein^–1^ for 222 and 254 nm, respectively),^17,19^ and most organic compounds exhibit stronger light absorption at 222 nm than at 254 nm.^20−22^

Since the discovery of trihalomethanes such as chloroform as byproducts in chlorine-based water disinfection in the 1970s,^23,24^ the need to reduce the risk of disinfection byproducts (DBPs) in drinking water is well recognized. Numerous studies have demonstrated that many DBPs are potent cytotoxicants, genotoxicants, and carcinogens.^25−28^ Based on the health findings, regulations are in place in the United States to limit the levels of DBPs in drinking water: the total concentration of four chlorinated and brominated trihalomethanes (THMs) at 80 μg·L^–1^, the total concentration of five chlorinated and brominated haloacetic acids (HAAs) at 60 μg·L^–1^, bromate at 10 μg·L^–1^, and chlorite at 1000 μg·L^–1^.^29^ However, the regulated DBPs represent only a small fraction of the >700 known DBPs.^30^ In recent years, research has increasingly focused on unregulated DBPs, particularly nitrogenous DBPs (N-DBPs), such as haloacetonitriles (HANs) and haloacetamides (HAMs), which contain one- and two-carbon atoms with nitrogenous functional groups.^30^ These N-DBPs have attracted significant attention due to their markedly higher genotoxicity and cytotoxicity compared to conventional DBPs, raising serious public health concerns.^31^ Their unique chemical characteristics and formation pathways necessitate further investigation to understand their occurrence, risk, and potential treatment strategies.

UV irradiation is a useful way to mitigate DBPs in water. For instance, a UV system was first employed in a swimming pool in Denmark in 1976, and it is estimated that there are currently 1000–2000 installations of UV systems in public swimming pools across Europe.^32^ In our study, we selected UV irradiation due to its proven effectiveness in both disinfection and DBP degradation, as demonstrated by recent investigations. The photolysis of DBPs by UV irradiation has been reported by many studies. For example, Hu et al.^33^ investigated the photodegradation of iodinated DBPs, triiodomethane, diiodoacetamide and triiodoacetic acid, using LPUV with a fluence rate of 8.88 × 10^–4^ Einstein·m^–2^·s^–1^, reporting the decay rate constants of 1.0 × 10^3^, 8.3 × 10^2^, and 7.6 × 10^2^ m^2^·Einstein^–1^ for these respective DBPs. Zhang et al.^34^ investigated the degradation of 40 different halogenated DBPs using LPUV at a fluence rate of 3.19 × 10^–6^ Einstein·L^–1^·s^–1^, resulting in decay rate constants ranging from 2.3 × 10^–4^ to 3.0 × 10^–4^ cm^2^·mJ^–1^. While there has been extensive research on LPUV, to date information is still scarce regarding the degradation of DBPs by using KrCl* excimer lamps. Given that far-UVC (e.g., 222 nm) offers higher photon energy, it can induce more efficient electronic transitions and generate reactive species more effectively, potentially leading to faster and more effective DBP degradation.

Furthermore, constituents in real water matrices could pose significant impacts on the application of KrCl* excimer lamps in water treatment. Anions such as chloride, sulfate, and bicarbonate/carbonate likely have small to minimal effects, due to their low light absorption.^35^ In contrast, nitrate and dissolved organic matter (DOM) have high absorptivity at 222 nm,^35^ hence a significant potential to compete with DBPs for UV photons. Meanwhile, reactive species, such as hydroxyl radical, reactive nitrogen species, and photoinduced reactive intermediates (e.g., ^1^O_2_ and ^3^DOM*), can be produced under 222 nm irradiation of nitrate and/or DOM,^22^ and these species could counteract the light-screening effect caused by water matrix. So far, few studies have investigated the impacts of water matrix on the photolysis of DBPs under 222 nm irradiation.

The objective of this study was to evaluate the photodegradation of DBPs, particularly HANs and HAMs, in water using a KrCl* excimer lamp emitting at 222 nm. A total of 12 nitrogenous DBPs were investigated to represent two types of halogen substitution (chloro- and bromo-) and two types of N-containing functional groups (acetonitrile and acetamide). First, the photolysis of each DBP at 222 nm was investigated. To probe mechanistic insight for the impacts of DBP structures on the photolysis at 222 nm, molar absorptivity, quantum yield, and electronic parameters (*E*_HOMO_, *E*_LUMO_ and others) of the DBPs were determined and utilized to assess correlations with the photolysis rates of DBPs. Then, the photodegradation of selected DBPs was compared between 222 and 254 nm wavelengths. Finally, the impacts of water matrix (nitrate and DOM) on DBP photolysis at 222 nm were evaluated in representative conditions.

## 2 Materials and Methods

### 2.1 Chemicals

Selected 12 DBPs were monochloroacetonitrile (MCAN), dichloroacetonitrile (DCAN), trichloroacetonitrile (TCAN), monochloroacetamide (MCAM), dichloroacetamide (DCAM), trichloroacetamide (TCAM), monobromoacetonitrile (MBAN), dibromoacetonitrile (DBAN), tribromoacetonitrile (TBAN), monobromoacetamide (MBAM), dibromoacetamide (DBAM) and tribromoacetamide (TBAM). The structures and properties of DBPs are provided in the Supporting Information (SI) Table S1. All individual DBP compounds were purchased at the highest purity from MilliporeSigma (St. Louis, MO) and TCI Chemical (Tokyo, Japan). 1,2-Dibromopropane (97%), sodium sulfate (≥99%), sodium nitrate (≥99%), sodium carbonate (≥99%), sodium chloride (≥99%), potassium iodide (≥99%), potassium iodate (≥99%) and *tert*-butyl methyl ether (MtBE) (>99.8%) were purchased from MilliporeSigma (St. Louis, MO). The mixture of fulvic acids was obtained from the International Humic Substances Society (IHSS). Reagent-grade deionized (DI) water (>18 mΩ-cm) was generated from a Milli-Q Nanopure water purification system (Billerica, MA).

### 2.2 Experimental Procedures

#### 2.2.1 UV Photolysis

Solutions containing 100 μg·L^–1^ DBP and 10 mM phosphate buffer (pH 6.9) were prepared in DI water. UV photolysis reaction was conducted in a glass reactor with 80 mL solution at room temperature (25 °C) under a bench-scale UV collimated beam apparatus with a KrCl* lamp (SI Figure S1(A)). To provide a comparison, selected experiments were carried out at 254 nm using a 4-W LPUV lamp (TUV4W, Philips) in a 100 mL cylindrical quartz reactor which has been well characterized in previous studies.^17,36−38^ The LPUV light was emitted from one side (SI Figure S1(B)). The solution was continuously stirred using a magnetic stirrer. Note that the hydrolysis of DBPs in DI water remained minimal for 60 min, which aligns with findings from previous studies (*k*_hydroysis_: HANs:^39^ 1.2 × 10^–6^ – 3.9 × 10^–4^ M^–1^·s^–1^; MBAM:^40^ 2.78 × 10^–7^ M^–1^·s^–1^; TBAM:^40^ 2.78 × 10^–7^ M^–1^·s^–1^).

To evaluate the effects of water matrix, selected experiments were conducted using DCAM in the solution containing 2.0–5.0 mg·L^–1^ fulvic acids and 1.0–10.0 mg·L^–1^ nitrate. *Tert*-butyl alcohol (TBA) ([TBA]_0_ = 1 mM) was used as a scavenger to assess the contribution of ^•^OH to DBP decay in the presence of nitrate and fulvic acids. Note that 1 mM TBA has zero absorption at 222 nm, ensuring that it does not interfere with the photon absorption of DBPs. Additionally, photodecay of DCAM was investigated in a simulated real water containing 0.679 mg·L^–1^ (as N) of nitrate, 2.56 mg·L^–1^ fulvic acids, 90 mg·L^–1^ carbonate, 40 mg·L^–1^ chloride, 55 mg·L^–1^ sulfate and 10 mM phosphate (pH 6.9). A DBP concentration of 100 μg·L^–1^ was chosen for mechanistic experiments, even though actual levels can vary widely with source water, treatment processes, and seasonal variation.

To assess the effect of oxygen, the photolysis of TCAM was evaluated in the presence and absence of oxygen for comparison. To achieve anoxic conditions, a desired volume of 10 mM phosphate buffer (pH 6.7) was purged with nitrogen (N_2_) gas for at least 45 min before being quickly transferred to a Petri dish reaction vessel and spiked with TCAM, and a gentle N_2_ gas stream was supplied continuously across the surface of the solution during the experimental run. The oxic solution was prepared using a 10 mM phosphate buffer (pH 6.7) that had been stirred in an open beaker for 24 h prior to the photolysis experiments to ensure the dissolved oxygen levels in equilibrium with the atmosphere. Oxic and anoxic runs were conducted in duplicates.

The UV fluence rate (*I*(λ)) was determined as 3.14 × 10^–6^ Einstein·L^–1^·s^–1^ and 2.23 × 10^–6^ Einstein·L^–1^·s^–1^ for the KrCl* lamp and LPUV setups, respectively, using KI/KIO_3_ actinometry.^41^ Briefly, the concentrations of I_3_^–^, which is a photoproduct, were measured using molar absorption coefficient (ε = 27636 M^–1^·cm^–1^ in 0.6 M KI/0.1 M KIO_3_ solution) at wavelength 352 nm,^42^ and the UV fluence rate (*E*(λ) in Einstein·cm^–2^·s^–1^) was determined by the eq 1).
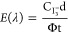
1where C_I_3_^–^_ is the concentration of I_3_^–^ (M), Φ is the quantum yield of I_3_^–^ (0.94 and 0.72 at 222 and 254 nm, respectively),^41^ d is the effective light path length (1.0 cm), and t is time (sec).

The molar absorption coefficients (ε, M^–1^·cm^–1^) of DBPs were determined at 222 and 254 nm in pH 6.8 solution buffered with 10 mM phosphate. The quantum yield (Φ) of DBPs was calculated using molar absorption coefficients at 222 and 254 nm and effective path lengths (2.3 cm for 222 nm and 3.5 cm for 254 nm). The effective path length in the KrCl* lamp setup was determined by dividing the solution’s volume by the surface area of crystallizing dish (radius = 3.3 cm). The effective path length in the LPUV lamp setup with a cylindrical reactor was determined using a previously described approach.^17,37,38^

All experiments were conducted in duplicate. The data presented includes the average and standard deviation of the duplicate measurements.

#### 2.2.2 DBP Analysis

DBPs were solvent-extracted using MtBE. Sample solutions (2 mL) were spiked with 40 μg·L^–1^ 1,2-dibromopropane, an internal standard, and mixed with 2 mL MtBE and 2.0 g sodium sulfate. Concentrations of DBPs in the MtBE layer were measured by gas chromatography (Hewlett-Packard 6890 GC) equipped with an electron capture detector (ECD) and a HP-5 column. The GC-ECD method employed the following parameters: 1–3 μL splitless injection at 160 °C; column temperature was held at 33 °C for 5 min, then raised to 40 °C at 7 °C per min and held for 5 min, and then raised to 120 °C at 15 °C per min and held for 1 min, and then raised to 250 °C at 25 °C per min and held for 1 min; ECD temperature was 290 °C, and the makeup gas was nitrogen with a constant flow rate of 18.8 mL·min^–1^.

### 2.3 Quantum Chemical Parameters Calculation

Quantum chemical parameters, including the energy of lowest unoccupied molecular orbital (*E*_LUMO_), the highest occupied molecular orbital (*E*_HOMO_), electronegativity (χ), electrophilicity index (ω), ionization potential (*IE*), electron affinity (*EA*), hardness (*η*), and softness (*σ*), of DBPs were determined using Density Functional Theory (DFT) calculations. All calculations utilized the atom-pairwise dispersion correction with the Becke-Johnson damping scheme (D3BJ). For all the simulated molecules, the B3LYP+D3^43−47^/6–311++G**^48−50^ basis set was used for geometry optimizations and frequency calculations. All calculations were conducted using the Orca electronic structure package (Version 4.2.1). Quantum chemical parameters of DBP compounds are listed in SI Table S2.

### 2.4 Statistical Analysis

Correlation analysis of DBP decay rate constants with several descriptors of DBPs, including absorption coefficient, quantum yield, quantum chemical parameters, and *E*_LUMO_-*E*_HOMO_, was conducted using PEARSON and TDIST functions in Microsoft Excel. Descriptors showing correlation coefficients (*r*) above 0.5 and *p*-value less than 0.05 were considered statistically significantly correlated with DBP decay. Subsequently, additional linear regression analyses were conducted when a descriptor demonstrated a strong correlation with the DBP decay rate.

## 3 Results and Discussion

### 3.1 UV Photolysis of Halogenated DBPs

The photolysis of 12 DBPs at 222 nm was investigated individually for 30 min irradiation, and the overall decay of parent DBPs was quantified ([DBP]_decay,%_ in %) at 30 min. The photolysis of DBPs over time followed pseudo-first-order kinetics (R^2^ > 0.92), and the decay rate constant was obtained by calculating the slope of ln *C*_t_/C_0_ against time or fluence. The rate constant was denoted as *k* (in sec^–1^, L·Einstein^–1^, or cm^2^·Einstein^–1^). [DBP]_decay,%_ and *k* are summarized in Table 1. Among the DBPs, the photolysis of MCAN is the slowest (*k* = 5.85 × 10^–5^ s^–1^ or 1.86 × 10^1^ L·Einstein^–1^; [DBP]_decay,%, 30 min_ = 5.2%), while the photolysis of TBAM is the fastest (*k* = 1.18 × 10^–2^ s^–1^ or 3.74 × 10^3^ L·Einstein^–1^; [DBP]_decay,%, 30 min_ = 100%). Overall, the presence of halogen substituents significantly influenced the degree and rate of photodecay of DBPs. More highly halogenated DBPs within the same DBP class exhibited a greater decay and higher rate constants. Within the same DBP class, bromo-DBPs showed a greater degree of decay and higher rate constants in comparison with chloro-DBPs. The observed trend in decay rates is consistent with the trend in absorption coefficients (ε), and the correlation between *k* and ε is further discussed in Section 3.3.

**Table 1:** Decay % and Rate Constants (*k*) for Photolysis of DBPs at 222 nm

				fluence-based rate constant (*k*)	
DBP compound	formula	[DBP]_decay,%_^a^	time-based rate constant (*k*; sec^–1^)	L·Einstein^–1^	cm^2^·Einstein^–1^^b^
monochloroacetonitrile (MCAN)	ClH_2_C–CN	5.2	(5.85 ± 0.87) × 10^–5^	(1.86 ± 0.28) × 10^1^	(7.96 ± 1.18) × 10^3^
dichloroacetonitrile (DCAN)	Cl_2_HC–CN	55.2	(4.78 ± 0.38) × 10^–4^	(1.52 ± 0.12) × 10^2^	(6.50 ± 0.51) × 10^4^
trichloroacetonitrile (TCAN)	Cl_3_C–CN	93.8	(1.43 ± 0.21) × 10^–3^	(4.56 ± 0.68) × 10^2^	(1.95 ± 0.29) × 10^5^
monochloroacetamide (MCAM)	ClH_2_C–CONH_2_	59.6	(8.93 ± 0.82) × 10^–4^	(2.84 ± 0.26) × 10^2^	(1.22 ± 0.12) × 10^5^
dichloroacetamide (DCAM)	Cl_2_HC–CONH_2_	92.7	(2.84 ± 0.26) × 10^–3^	(9.04 ± 0.84) × 10^2^	(3.86 ± 0.36) × 10^5^
trichloroacetamide (TCAM)	Cl_3_C–CONH_2_	99.2	(4.96 ± 0.28) × 10^–3^	(1.58 ± 0.91) × 10^3^	(6.75 ± 0.51) × 10^5^
monobromoacetonitrile (MBAN)	BrH_2_C–CN	42.9	(3.28 ± 0.21) × 10^–4^	(1.04 ± 0.07) × 10^2^	(4.46 ± 0.28) × 10^4^
dibromoacetonitrile (DBAN)	Br_2_HC–CN	98.4	(3.87 ± 0.21) × 10^–3^	(1.23 ± 0.11) × 10^3^	(5.27 ± 0.28) × 10^5^
tribromoacetonitrile (TBAN)	Br_3_C–CN	96.3	(8.73 ± 0.11) × 10^–3^	(2.78 ± 0.35) × 10^3^	(1.19 ± 0.15) × 10^6^
monobromoacetamide (MBAM)	BrH_2_C–CONH_2_	61.5	(1.05 ± 0.41) × 10^–3^	(3.34 ± 0.13) × 10^2^	(1.43 ± 0.48) × 10^5^
dibromoacetamide (DBAM)	Br_2_HC–CONH_2_	93.5	(8.14 ± 0.10) × 10^–3^	(2.59 ± 0.53) × 10^3^	(1.11 ± 0.12) × 10^6^
tribromacetamide (TBAM)	Br_3_C–CONH_2_	100	(1.18 ± 0.12) × 10^–2^	(3.74 ± 0.42) × 10^3^	(1.60 ± 0.14) × 10^6^

a Degree of DBP decay for 30 min.

b Fluence-based *k* (in cm^2^·Einstein^–1^) was calculated by dividing the fluence-based *k* (in L·Einstein^–1^) with effective path length (*l* = 2.3 cm).

**Figure 1:**
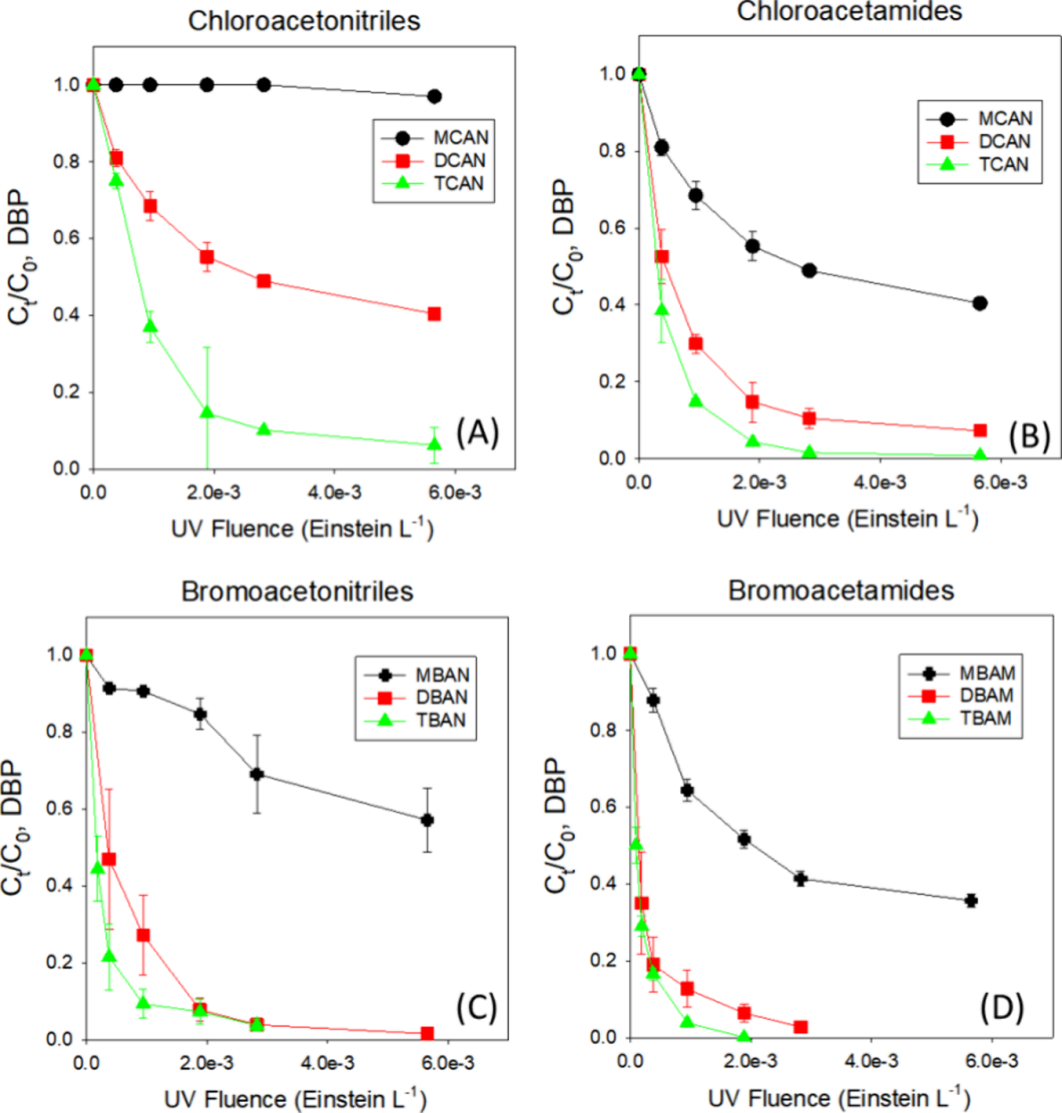
Photolysis of nitrogenous DBPs at 222 nm in reagent water. (A) chloroacetonitriles, (B) chloroacetamides, (C) bromoacetonitriles, and (D) bromoactamides. Reaction condition: [DBP]_0_ = 100 μg·L^–1^, pH = 6.9, 10 mM phosphate. DBPs: MCAN-monochloroacetonitrile, DCAN-dichloroacetonitrile, TCAN-trichloroacetonitrile, MCAM-monochloroacetamide, DCAM-dichloroacetamide, TCAM-trichloroacetamide, MBAN-monobromoacetonitrile, DBAN-dibromoacetonitrile, TBAN-tribromoacetonitrile, MBAM-monobromoacetamide, DBAM-dibromoacetamide TBAM-tribromoacetamide.

Specifically, the photodecay rate constants (*k* in sec^–1^ at 222 nm) of chloroacetonitriles followed the trend of TCAN (1.43 × 10^–3^) > DCAN (4.78 × 10^–4^) > MCAN (5.85 × 10^–5^), and [DBP]_decay,%,30 min_ ranged from 93.8% to 5.2% (Table 1 and Figure 1(A)). The photodecay rate constants of chloroacetamides followed the order of TCAM (4.96 × 10^–3^) > DCAM (2.84 × 10^–3^) > MCAM (8.93 × 10^–4^), and [DBP]_decay,%, 30 min_ ranged from 99.2% to 59.6% (Table 1 and Figure 1(B)). Chloroacetamides photodecayed 3–15 times as rapidly as chloroacetonitriles with the same number of chlorine substitutions. From one to three chlorine substitutions, TCAN photodecayed 24 times as fast as MCAN, while TCAM photodecayed 5.5 times as fast as MCAM.

The photodecay rate constants (*k* in sec^–1^ at 222 nm) of bormoacetonitriles were TBAN (8.73 × 10^–3^) > DBAN (3.87 × 10^–3^) > MBAN (3.28 × 10^–4^), and [DBP]_decay,%, 30 min_ ranged from 98.4% to 42.9% (Table 1 and Figure 1(C)). The photodecay rate constants of bromoacetamides were TBAM (1.18 × 10^–2^) > DBAM (8.14 × 10^–3^) > MBAM (1.05 × 10^–3^), and [DBP]_decay,%, 30 min_ ranged from 100% to 61.5% (Table 1 and Figure 1(D)). Bromoacetamides photodecayed 1–3 times as rapidly as bromoacetonitriles with the same number of bromine substitutions. From one to three bromine substitutions, TBAN photodecayed 26 times as fast as MBAN, while TBAM photodecayed 11 times as fast as MBAM. With the same number of halogen substitutions, bormoacetonitriles photodecayed 6–8 times as rapidly as chloroacetonitriles, while bromoacetamides photodecayed 1–3 times as rapidly as chloroacetamides.

### 3.2 Impacts of Water Matrix and Oxygen

To assess the influence of water matrix on photolysis, DCAM was chosen as a representative DBP compound. As a moderately halogenated compound, DCAM represents the range of halogenation levels (mono-, di-, and trihalogenated) observed among the DBPs in this study. The effects of nitrate and DOM were examined. In these experiments, the fluence rate was corrected by the water factor of nitrate and fulvic acids as follows:^51^
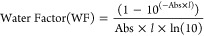
2
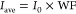
3where Abs is the absorbance of solution containing nitrate and/or fulvic acids (cm^–1^) at 222 nm, *l* is effective path length (cm), *I*_0_ and *I*_ave_ are the fluence rate before and after correction for solution absorbance, respectively (Einstein·L^–1^·s^–1^). The fluence rate was observed to decrease by 1.13–3.35 times in the presence of 1.0–10.0 mg·L^–1^ of nitrate and 1.14–1.34 times in the presence of 2.0–5.0 mg·L^–1^ of fulvic acids, compared to that in the absence of water matrix constituents. This decrease in fluence rate can be attributed to the light-screening effect caused by the presence of nitrate and fulvic acids. Corrected fluence rates are listed in SI Table S3.

#### 3.2.1 Nitrate

Nitrate exhibits substantial light absorption at 222 nm, characterized by high molar absorption coefficient (ε = 2700 M^–1^·cm^–1^).^52^ Strong light absorption by nitrate could result in light-screening effects on the DBPs. Indeed, the light transmittance (T%) of the solution was decreased to 25% with 10 mg·L^–1^ nitrate. The first-order rate constant (*k* in sec^–1^) for DCAM decay at 222 nm was reduced by 42%–67% in the presence of 1.0–10.0 mg·L^–1^ nitrate (SI Table S4).

**Figure 2:**
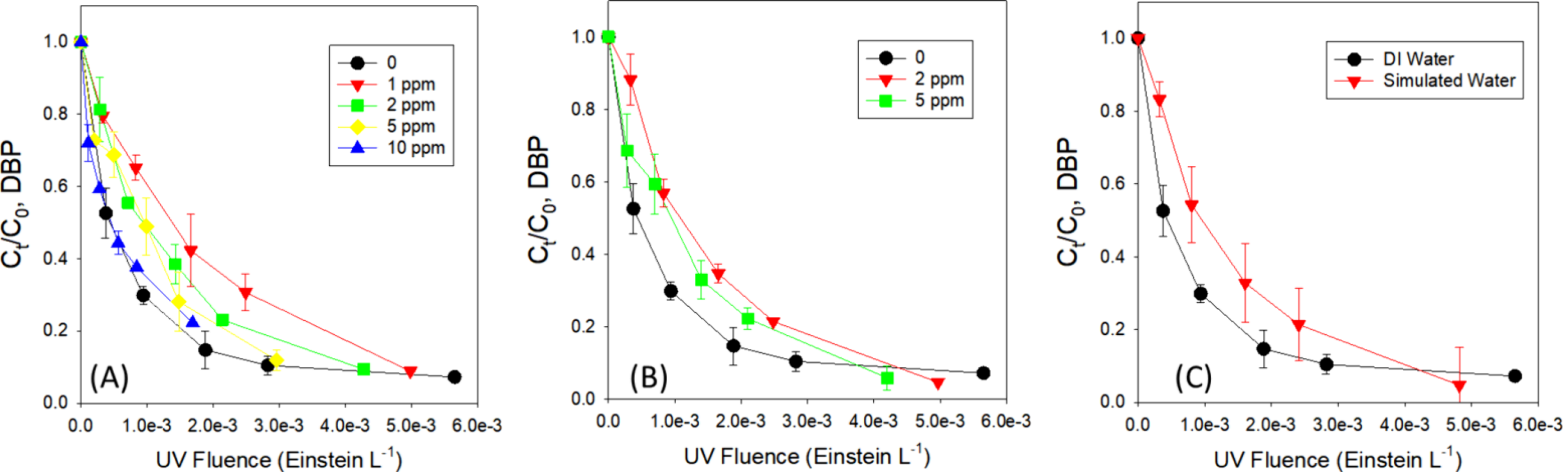
Photolysis of dichloroacetamide (DCAM) at 222 nm in water containing (A) nitrate and (B) fulvic acids, and (C) in simulated water. Fluence is shown based on the corrected fluence rate (I_ave_) for different matrices. Reaction condition: [DBP]_0_ = 100 μg·L^–1^, pH = 6.9, 10 mM phosphate for (A) and (B). For (c), [nitrate]_0_ = 0.679 mg·L^–1^ as N, [fulvic acids]_0_ = 2.56 mg·L^–1^, [carbonate]_0_ = 90 mg·L^–1^, [chloride]_0_ = 40 mg·L^–1^, [sulfate]_0_ = 55 mg·L^–1^, 10 mM phosphate, pH 6.9.

To assess the impact of light screening caused by nitrate, the fluence-based rate constant was recalculated based on *I*_ave_ which takes into account the water factor. The adjusted fluence-based rate constant in the presence of 1.0 mg·L^–1^ nitrate remained 46% lower than that observed in buffered water (Figure 2(A) and SI Table S4). However, with greater concentrations of nitrate from 1.0 to 10.0 mg·L^–1^, the adjusted fluence-based rate constant for DCAM decay exhibited an increase, with the rate constant ascending from 4.92 × 10^2^ L·Einstein^–1^ to 1.31 × 10^3^ L·Einstein^–1^. These results suggest the likelihood of reactive species, such as hydroxyl radical and reactive nitrogen species (RNS), generated from nitrate photolysis under 222 nm irradiation, contributing to DBP decay. For example, Payne et al.^22^ reported the formation of ^•^OH through the photolysis of nitrate at 222 nm with a steady-state concentration of ^•^OH at 1.44 × 10^11^ M·cm^2^·mW^–1^. In this study, TBA quencher was used to assess the role of ^•^OH and found to have a minimal effect on DCAM decay (SI Figure S2(A)). This result suggests that RNS, rather than ^•^OH, could have a more significant impact on DBP decay. Previous studies by Xu et al.^53^ and Li et al.^54^ documented the formation of RNS species (e.g., ^•^NO_2_, N_2_O_4_, ^•^NO, ONOO^–^) from nitrate under 222 nm. Interestingly, we also observed significant increase of nitrate decay and nitrite (NO_2_^–^) formation by the presence of TBA (SI Figure S2(B and C)). TBA can block the slow reaction of nitrate with ^•^OH (NO_3_^–^ + ^•^OH → ^•^NO_3_ + OH^–^; *k* < 1 × 10^5^ M^–1^·s^–1^),^55^ which can shift nitrate decay toward direct photolysis and RNS pathways. Moreover, as nitrite is a byproduct of decomposition of N_2_O_4_, TBA can hinder the sink of nitrite by blocking the reaction of nitrite with ^•^OH (NO_2_^–^ + ^•^OH → ^•^NO_2_ + OH^–^; *k* = 1.0 × 10^5^ M^–1^·s^–1^).^53^ The fact that DBP decay was not changed by the presence of TBA also implies that RNS contributed to DBP decay.

#### 3.2.2 Organic Matter

Fulvic acids were selected as a representative DOM component (Figure 2(B) and SI Table S4). The light transmittance of the solution was decreased to 77% with 5.0 mg·L^–1^ fulvic acids. The first-order rate constant (*k* in sec^–1^) for DCAM decay at 222 nm was reduced by 39% in the presence of 2.0–5.0 mg·L^–1^ fulvic acids.

The adjusted fluence-based rate constant for DCAM decay at 222 nm, considering the water factor for fluence rate, was found to decrease by 31% in the presence of 2.0 mg·L^–1^ fulvic acids and by 17% in the presence of 5.0 mg·L^–1^ fulvic acids, compared to the condition without fulvic acids. This finding suggests that factors other than light screening by fulvic acids also played a role in affecting DBP decay under 222 nm irradiation. Similar to nitrate, a greater concentration of fulvic acids showed a higher adjusted fluence-based rate constant, indicating the potential for indirect photolysis of DBP through reactive species (e.g., ^•^OH, ^1^O_2_,^3^DOM*) that may be formed from fulvic acids under 222 nm irradiation. In this study, ^•^OH was found to play a minimal role, as evidenced by the negligible impact of TBA on DCAM decay (SI Figure S3), suggesting that other reactive species, such as ^1^O_2_ and ^3^DOM*, had a more significant role in DBP photodegradation in the presence of fulvic acids.

#### 3.2.3 Simulated Water

The photodecay of DCAM was further investigated under simulated water condition containing 0.679 mg·L^–1^ (as N) of nitrate, 2.56 mg·L^–1^ fulvic acids, 90 mg·L^–1^ carbonate, 40 mg·L^–1^ chloride, 55 mg·L^–1^ sulfate, and 10 mM phosphate (pH 6.9) (Figure 2(C) and SI Table S4). The first-order rate constant (*k* in sec^–1^) for DCAM decay at 222 nm decreased by 37% in the simulated water compared to phosphate-buffered water. Furthermore, the adjusted fluence-based rate constant in simulated water remained 28% lower than that observed in buffered water.

#### 3.2.4 Effect of Oxygen

In the consideration that dissolved oxygen may absorb photons at 222 nm, albeit very weak (ε = 7.81 M^–1^·cm^–1^ at 24 °C under 215 nm),^56^ which may lead to reactive oxygen species that could contribute to the decay of DBPs, the potential impact of dissolved oxygen was evaluated. TCAM in phosphate-buffered water was selected as a representative and subjected to 222 nm UV radiation in the presence and absence of dissolved oxygen. As shown in SI Figure S4, there was no significant difference in the TCAM photolysis rate with and without dissolved oxygen present. Hence, reactive species generated by the absorption of UV radiation by dissolved oxygen was expected to play a very minor role, if any, in DBP photolysis by 222 nm UV radiation.

### 3.3 Mechanistic Insights for Photodecay of DBPs at 222 nm

To assess the mechanisms of how DBPs’ structures affect their photodecay under 222 nm irradiation, several descriptors, including molar absorption coefficient, quantum yield, *E*_HOMO_, and *E*_LUMO_, electronegativity (*χ*), electrophilicity index (*ω*), ionization potential (*IE*), electron affinity (*EA*), hardness (*η*), and softness (*σ*) were determined. Then, the correlations between the degree and rate of photodecay and descriptors were evaluated. These descriptors were chosen because they provide complementary insights into the electronic and photophysical properties that govern how compounds interact with UV light and subsequently decay.

#### 3.3.1 Molar Absorptivity and Quantum Yield of DBPs

As determined, the molar absorption coefficients (ε, M^–1^·cm^–1^) of DBPs at 222 nm range from 5.29 M^–1^·cm^–1^ for MCAN to 3.28 × 10^3^ M^–1^·cm^–1^ for TBAM (Table 2). The molar absorption coefficients of DBPs at 222 nm are at least 2.3 times greater than those at 254 nm (Table 2 and SI Table S5). The molar absorptivity at 222 nm is clearly influenced by the presence of halogen substituents on DBPs, with the trend showing higher values for bromo-DBPs compared to chloro-DBPs. Specifically, the molar absorptivity of bromo-DBPs is more than 3.4 times greater than that of chloro-DBPs within the same DBP class (Table 2). Hence, bromo-DBPs have a greater ability to absorb light at 222 nm, leading to enhanced photodegradation under KrCl* excimer lamp irradiation.

**Table 2:** Molar Absorption Coefficient (ε), Quantum Yield (Φ), *E*_HOMO_, *E*_LUMO_ for DBPs at 222 and 254 nm

Wavelength	DBP compound	ε (M^–1^·cm^–1^)	Φ	*E*_HOMO_	*E*_LUMO_	*E*_LUMO_–*E*_HOMO_
222 nm	MCAN	5.29	6.54 × 10^–1^	–8.987	–1.475	7.512
	DCAN	1.98 × 10^1^	1.43	–9.206	–1.854	7.352
	TCAN	5.05 × 10^1^	1.67	–9.198	–2.156	7.042
	MCAM	1.33 × 10^2^	3.97 × 10^–1^	–7.523	–0.426	7.097
	DCAM	3.84 × 10^2^	4.37 × 10^–1^	–7.826	–1.247	6.579
	TCAM	9.74 × 10^2^	3.01 × 10^–1^	–8.036	–1.539	6.497
	MBAN	3.95 × 10^2^	4.91 × 10^–2^	–8.400	–1.996	6.404
	DBAN	1.18 × 10^3^	1.95 × 10^–1^	–8.433	–2.560	5.872
	TBAN	2.55 × 10^3^	2.02 × 10^–1^	–8.332	–3.047	5.286
	MBAM	8.08 × 10^2^	7.66 × 10^–2^	–7.475	–0.927	6.548
	DBAM	2.16 × 10^3^	2.23 × 10^–1^	–7.717	–1.792	5.925
	TBAM	3.28 × 10^3^	2.12 × 10^–1^	–7.758	–2.464	5.294
254 nm	MCAN	7.56 × 10^–1^	3.15 × 10^–1^	–8.987	–1.475	7.512
	DCAN	2.84	4.24 × 10^–1^	–9.206	–1.854	7.352
	TCAN	1.44 × 10^1^	4.77 × 10^–1^	–9.198	–2.156	7.042
	MCAM	3.74	2.53 × 10^–1^	–7.523	–0.426	7.097
	DCAM	1.28 × 10^1^	3.12 × 10^–1^	–7.826	–1.247	6.579
	TCAM	3.25 × 10^1^	2.04 × 10^–1^	–8.036	–1.539	6.497
	MBAN	7.80 × 10^1^	1.09 × 10^–2^	–8.400	–1.996	6.404
	DBAN	5.13 × 10^2^	1.43 × 10^–1^	–8.433	–2.560	5.872
	TBAN	1.08 × 10^3^	1.08 × 10^–1^	–8.332	–3.047	5.286
	MBAM	7.58 × 10	5.70 × 10^–2^	–7.475	–0.927	6.548
	DBAM	2.78 × 10^2^	1.80 × 10^–1^	–7.717	–1.792	5.925
	TBAM	1.01 × 10^3^	1.58 × 10^–1^	–7.758	–2.464	5.294

The degradation of DBPs by UV photolysis can be described using the Beer–Lambert Law. The eq 4 applies to photolysis in a clean water matrix with a low concentration of DBPs in this work. The quantum yield of DBPs under UV irradiation was calculated using the eq 5.
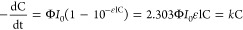
4
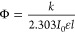
5where C is the concentration of DBPs (mol·L^–1^), t is the reaction time (sec), Φ is the quantum yield of DBPs (mol·Einstein^–1^), *I*_0_ is the fluence rate of the incident light at 222 nm (3.14 × 10^–6^ Einstein·L^–1^·s^–1^), ε is the molar absorption coefficient of DBP at 222 nm (M^–1^·cm^–1^), *l* is the effective path length (2.3 cm in this study), and *k* is the time-based rate constant of DBP decay at 222 nm (sec^–1^).

Herein, the quantum yields (mol·Einstein^–1^) of 12 DBPs at 222 nm are reported for the first time, with values ranging from 0.049 to 1.43. The quantum yields of DBPs at 222 nm are at least 1.2 times greater than those at 254 nm (Table 2 and SI Table S5). For those DBPs whose quantum yields are greater than one (DCAN and TCAN), self-sensitization likely occurred in their photolysis under 222 nm irradiation.

**Figure 3:**
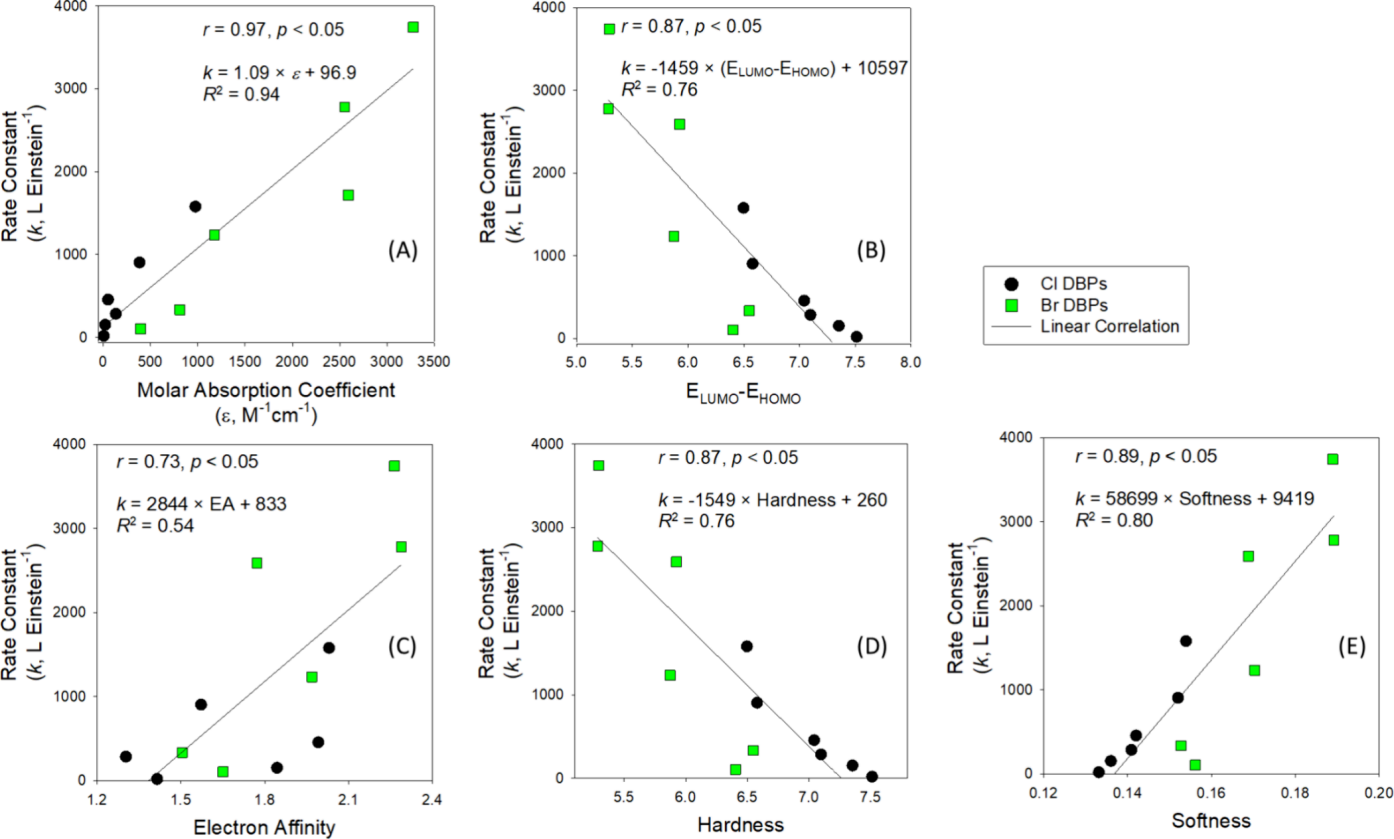
Relationship between rate constants (*k*, L·Einstein^–1^) for DBP photodegradation at 222 nm with (A) molar absorption coefficient (ε), and (B) *E*_LUMO_–*E*_HOMO_, (C) Electron affinity, (D) Hardness, and (E) Softness.

When exposed to UV irradiation, organic molecules are activated by photons to transition to excited states, followed by further reactions to generate photoproducts.^17^ The molar absorption coefficient and quantum yield should highly influence the photolysis of organic molecules. A strong positive linear correlation was observed between the rate constants for DBP decay at 222 nm and the molar absorption coefficient (*r* = 0.95, *p* < 0.05): *k* (L·Einstein^–1^) = 1.09 × ε + 96.9, R^2^ = 0.94 (Figure 3(A)). This strong correlation indicates that DBPs with higher ε values absorb more photons, resulting in higher photolysis rate constants. In contrast, the quantum yield featured a poor correlation with the rate constant for DBP photodecay (*r* = 0.38, *p* > 0.05) (SI Figure S5(A)). A linear relationship (*r* = 1) existed between *k* and the product of ε and Φ (ε × Φ) according to eq 5.

#### 3.3.2 Quantum Chemical Parameters

The electronic influence of halogen substituents on DBPs may help explain the photodecay trends. Halogenated DBPs are capable of absorbing UV photons through electronic transitions (n → σ*) from the highest occupied molecular orbital (HOMO, a lone pair electron on a halogen atom) to the lowest unfilled molecular orbital (LUMO).^57,58^ The activation energy can be expressed by the energy gap between the ground state reactants and the excited transition state, *E*_LUMO_ - *E*_HOMO_.^58^ This energy gap is directly associated with chemical reactivity, influencing the degree of electron transfer in UV photolysis. DBPs with fewer halogenated species exhibit lower electronegativity compared to more halogenated analogues, which may result in a lower degradation rate. The replacement of a chlorine by a bromine lowers the *E*_LUMO_ but increases the *E*_HOMO_ (SI Table S2).

A significant negative linear correlation was observed between the rate constant for DBP decay at 222 nm and the *E*_LUMO_ - *E*_HOMO_ energy gap (*r* = 0.87, *p* < 0.05): *k* (L·Einstein^–1^) = −1459 × (*E*_LUMO_ - *E*_HOMO_) + 10597, R^2^ = 0.76 (Figure 3(B)). The significant negative correlation indicates that DBPs with smaller HOMO–LUMO energy gaps are more readily excited to reactive states, facilitating more efficient photochemical transformations and resulting in higher observed photodegradation rates. In comparison, *E*_LUMO_ and *E*_HOMO_ individually had a poor correlation with the rate constant for DBP decay at 222 nm (*r* = 0.37 and 0.56, respectively, *p* > 0.05) (SI Figure S5 (B,C)). Electron affinity, hardness, and softness demonstrated a correlation with the rate constants for DBP decay at 222 nm (Figure 3(C-E)); however, other parameters, including electronegativity, electrophilicity, and ionic potential, showed poor correlations (SI Figure S5 (D-F)). In addition, significant correlations were observed between the quantum yields of DBPs and *E*_HOMO_ (*r* = 0.76, *p* < 0.05), *E*_LUMO_ - *E*_HOMO_ (*r* = 0.61, *p* < 0.05) and ionization potential (*r* = 0.68, *p* < 0.05), while other parameters showed poor correlations (SI Figure S6 (A-F)). Overall, the electronic properties of DBP structures support the behaviors in light absorption and photodegradation quantum yield.

#### 3.3.3 Preliminary Transformation Product Analysis

The transformation products of selected DBPs, DBAM and TCAN, under 222 nm irradiation were analyzed. To ensure that the transformation products could be detected, higher concentrations of the DBPs were used in these experiments ([DBAM]_0_ = 175 μmol·L^–1^, [TCAN]_0_ = 54 μmol·L^–1^, pH = 6.9, 10 mM phosphate). For DBAM, MBAM ([MBAM] at 30 min = 13.5 μmol·L^–1^ for 30 min) and Br^–^ ([Br^–^] at 30 min = 394.8 μmol·L^–1^ for 30 min) were observed as transformation products during the photolysis process (SI Figure S7(A)). Total bromine remained nearly constant, with recoveries of 100–118%, while the concentration of Br^–^ steadily increased. This result indicates that the bromine in DBAM was predominantly transformed into Br^–^. In contrast, for TCAN, no less halogenated DBPs (i.e., MCAN and DCAN) were observed; only Cl^–^ ([Cl^–^] at 30 min = 55.8 μmol·L^–1^ at 30 min) was observed as a transformation product (SI Figure S7(B)). This differs from previous study^59^ conducted under vacuum UV (185 and 254 nm), which reported the formation of DCAN. Total chlorine decreased, with a recovery of 36% after 30 min-irradiation, suggesting the formation of unidentified products. Further studies should focus on identifying these unknown transformation products and investigate the wavelength-dependent differences in photolysis mechanisms to better understand the underlying reaction pathways.

### 3.4 Comparison with DBP Degradation under 254 nm Irradiation

**Figure 4:**
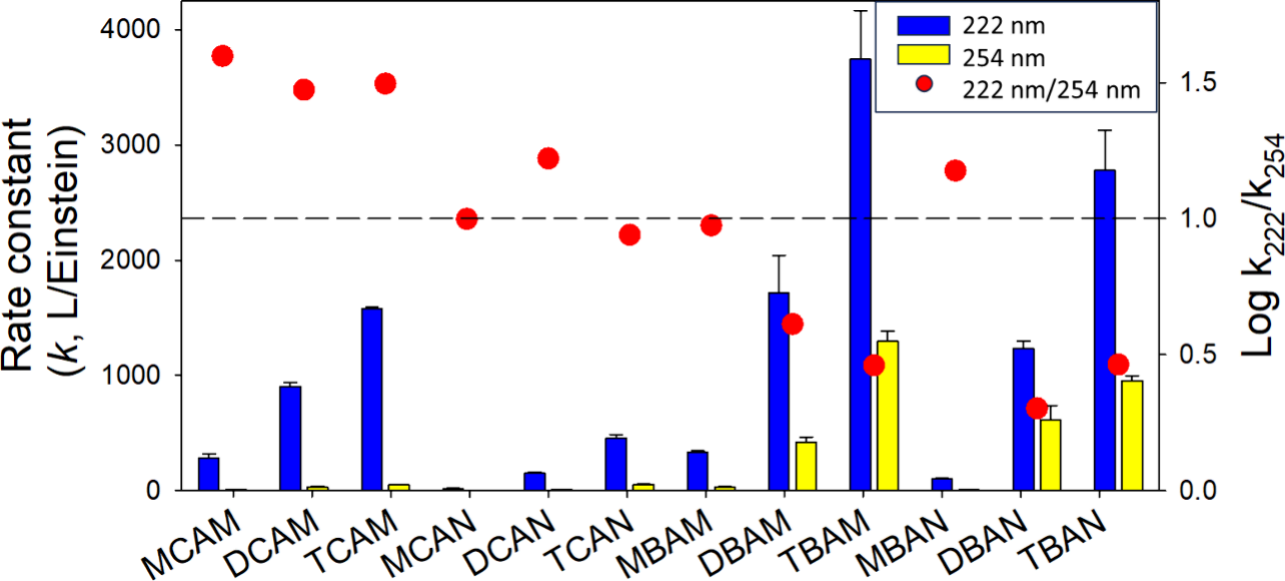
Photolysis rate constants (in L·Einstein^–1^) of DBPs at different irradiation wavelengths (222 and 254 nm).

The photodecay of 12 DBPs at 222 nm was compared to their decay at 254 nm under the same fluence and reaction conditions (Figure 4, SI Figure S8 and S9). The fluence-based rate constants (cm^2^·Einstein^–1^) for DBP decay at 222 nm were 3–56 times higher compared to the rate constants at 254 nm (SI Table S6). The quantum yields were also higher at 222 nm than at 254 nm by 1.2–4.5 times (Table 2 and SI Table S5). As mentioned above, the molar absorption coefficient of DBPs is significantly higher at 222 nm than at 254 nm by 2.4 to 35.1 times (Table 2). The stronger light absorption and higher quantum yield both result in intensified photoreactions and accelerated decay rates at 222 nm compared to 254 nm.

We calculated the fluence required to degrade half of DBP concentration (E_50%_) (SI Table S7). E_50%_ ranges from 2.33 × 10^2^ to 4.69 × 10^4^ mJ·cm^–2^ at 222 nm, whereas is 2.7–49 times higher at 254 nm from 8.94 × 10^2^ to 5.97 × 10^5^ mJ·cm^–2^. Although the current wall-plug efficiency of KrCl* excimer lamps (5–15%) is lower than that of LPUV lamp (30–38%),^60^ KrCl* excimer lamps deliver 2.7–49 times higher photodegradation rates and hence still hold the advantage. Using KrCl* excimer lamps, fluence less than 340 mJ·cm^–2^ can achieve 50% removal of brominated TBAM, DBAM and TBAN, while 550–970 mJ·cm^–2^ fluence is needed for TCAM, DBAN and DCAM, in clean water matrix. Removal of monobrominated or monochlorinated DBPs requires 2620–46,900 mJ·cm^–2^, much more difficult to achieve in practical conditions. Meanwhile, the toxicity of the N-DBPs is higher for bromo-DBPs than chloro-DBPs and with a higher degree of halogenation.^61,62^ Comparatively, a fluence of 894 mJ·cm^–2^ is needed to remove 50% of the most photolabile TBAM, whereas fluences in the range of thousands of mJ·cm^–2^ or higher are required for the other DBPs using LPUV lamps.

Similar to 222 nm, the rate constants for DBP decay at 254 nm exhibited a strong linear correlation with both the molar absorption coefficient (r = 0.86, *p* < 0.05) and the *E*_LUMO_ - *E*_HOMO_ energy gap (*r* = 0.87, *p* < 0.05) (SI Figure S10). Additionally, the quantum yields of DBPs at 254 nm were significantly correlated with *E*_HOMO_ (*r* = 0.59, *p* < 0.05) and with *E*_LUMO_ - *E*_HOMO_ (*r* = 0.63, *p* < 0.05) (SI Figure S10). These findings suggest that the photodecay mechanism remains largely consistent across both wavelengths.

## 4 Conclusions

This study demonstrated that mono-, di-, and tribrominated or chlorinated haloacetonitriles and haloacetamides exhibit a greater susceptibility to photolysis when exposed to 222 nm UV irradiation, compared to conventional 254 nm irradiation. At 222 nm, these nitrogenous DBPs photodegrade at 3–56 times faster fluence-based rate constants with 1.2–4.5 times higher quantum yields compared to 254 nm. More halogenation in the N-DBPs results in a greater and faster photodecay. Bromo-DBPs exhibit greater and faster decay than chloro-DBPs. The photodegradation of N-DBPs at 222 nm in clean water matrix is mainly direct photolysis, which depends critically on light absorption and photoexcitation and strongly correlates with molar absorptivity, the (*E*_LUMO_ - *E*_HOMO_) gap, electron affinity, hardness, and softness of DBPs. In real water matrices, nitrate and DOM at environmentally relevant concentrations can pose considerable light-screening effects due to their strong light absorption at 222 nm. However, the light-screening effect is countered by reactive species generated from photoreactions of nitrate and DOM contributing to DBP decay, leading to an overall moderate impact. Experimental results suggest that reactive species other than ^•^OH radicals likely play a more significant role in DBP decay. Calculations of fluence requirement to achieve 50% removal of DBPs indicate that KrCl* excimer lamps exhibit much better promise than LPUV lamps in the degradation of tri- and dibrominated or chlorinated N-DBPs. Future studies can focus on further assessing the photodegradation of other DBPs and the effects of water matrix constituents that generate reactive species on the decay pathways and products of DBPs at 222 nm irradiation.

## Supporting Information Available

Structures and chemical properties (Table S1); quantum chemical parameters used in this study for DBPs (Table S2); fluence rate with corrections using water factor (WF) (Table S3); decay % and rate constant (*k*) for DCAM photodegradation at 222 nm in the presence of water matrix constituent (Table S4); comparison of molar absorption coefficient (ε), quantum yield (Φ), *E*_HOMO_, and *E*_LUMO_ for DBPs at 222 and 254 nm (Table S5); rate constants (*k*) for photolysis of DBPs at 254 nm (Table S6); fluence required to achieve the removal of half of DBP concentration (E_50%_; mJ cm^2^) (Table S7); UV setup for experiments (Figure S1); TBA impacts on DCAM decay and nitrite formation in the presence of nitrate (Figure S2); TBA impacts on TCAN decay in the presence of fulvic acid (Figure S3); photolysis of TCAM in the presence and absence of oxygen (Figure S4); correlation between rate constants (*k*, L Einstein^–1^) for DBP photodegradation at 222 nm with quantum yield and electronic parameters (Figure S5); correlation between quantum yield with electronic parameters (Figure S6); transformation products of DBAM and TCAN (Figure S7); comparison of photolysis of chloro-DBPs at 222 and 254 nm (Figure S8); comparison of photolysis of bromo-DBPs at 222 and 254 nm (Figure S9); correlation between rate constants for DBP photodegradation or quantum yield of DBP at 254 nm with molar absorption coefficient and quantum chemical parameters (Figure S10) (PDF)
