# Supplementary material for: Photodegradation of Nitrogenous Disinfection Byproducts by Far-UVC Light at 222 nm
Source: ACS ES T Water. 2025 Apr 10;5(5):2619–29. doi: 10.1021/acsestwater.5c00156 (PMC12070416; doi:10.1021/acsestwater.5c00156)
Supplement: Supplementary file 1 — ew5c00156_si_001.pdf [file ew5c00156_si_001.pdf]

Supporting Information

for

Photodegradation of Nitrogenous Disinfection By-products by Far-UVC Light at 222 nm

Juhee Kim,[a,b,*] Xiaoyue Xin,[a] Ryan J. Kann,[c] Jiaqi Li,[a] Aidan S. Labrozzi,[a] Jiale Xu,[a,d] and Ching-Hua Huang[a,*]

[a] School of Civil and Environmental Engineering, Georgia Institute of Technology, Atlanta, GA 30332, United States

[b] Department of Civil, Environmental and Construction Engineering, University of Hawai`i at Mānoa, Honolulu, HI 96822, United States

[c] School of Biology, Georgia Institute of Technology, Atlanta, GA 30332, United States

[d] Department of Civil, Construction and Environmental Engineering, North Dakota State University, Fargo, North Dakota 58102, United States

*Corresponding Authors. Emails: ching-hua.huang@ce.gatech.edu (Ching-Hua Huang); juheek@hawaii.edu (Juhee Kim)

Number of Pages: 19

Number of Tables: 7

Number of Figures: 10

Contents

Tables

Table S1. Structures and chemical properties of DBPs.-- S3

Table S2. Quantum chemical parameters used in this study for DBPs.-------------------------- S4

Table S3. Fluence rate with corrections using water factor (WF) .------------------------------ S5

Table S4. Decay% and rate constant (*k*) for DCAM decay at 222 nm in the presence of water matrix constitutent.-- S6

Table S5. Comparison of molar absorption coefficient (ε), quantum yield (Φ), E_{HOMO}, E_{LUMO} for DBPs at 222 nm and 254 nm.-- S7

Table S6. Decay % and rate constants (*k*) for DBPs at 254 nm.--------------------------------- S8

Table S7. Fluecne required to achieve half of DBP concentration ($E_{50\%}$; mJ·cm^2).------------ S9

Figures

Figure S1. UV set-up for (A) KrCl* Excimer lamp emitting 222 nm and (B) LPUV lamp emitting 254 nm. ---S10

Figure S2. (A) Photolysis of dichloroacetamide (DCAM) at 222 nm in the presence and absence of TBA in the presence of nitrate. (B-C) Nitrite formation at 222 nm in the presence and absence of TBA in the presence of nitrate. --------------------------------------S11

Figure S3. Photolysis of dichloroacetamide (DCAM) at 222 nm in the presence and absence of TBA in the presence of fulvic acid. ---S12

Figure S4. Photolysis of tricloroacetamide (TCAM) at 222 nm in the presence and absence of oxygen. ---S13

Figure S5. Correlation between rate constants for DBP photodegradation at 222 nm with quantum yield and electronic parameters. ---S14

Figure S6. Correlation between quantum yield of DBP at 222 nm with electronic parameters. ---S15

Figure S7. Transformation products of (A) DBAM and (B) TCAN at 222 nm. ---------------S16

Figure S8. Comparison of photolysis of chloro-DBPs at different irradiation wavelengths (222-nm and 254-nm). ---S17

Figure S9. Comparison of photolysis of bromo-DBPs at different irradiation wavelengths (222-nm and 254-nm). ---S18

Figure S10. Correlation between rate constants for DBP photodegradation or quantum yield of DBP at 254 nm with molar absorption coefficient and quantum chemical parameters.---S19

Table S1. Structures and chemical properties of DBPs used in this study.

DBPs	log K_{ow}	water solubility (mg L^{-1}, 25 °C)	molecular weight (g mol^{-1})	chemical structure
monochloroacetonitrile (MCAN)	0.45	> 1×10^5	75.50	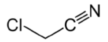
dichloroacetonitrile (DCAN)	0.29	3.4×10^4	109.94	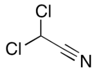
trichloroacetonitrile (TCAN)	2.09	7.2×10^2	144.38	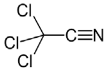
monochloroacetamide (MCAM)	-0.53	9.0×10^4	93.51	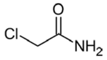
dichloroacetamide (DCAM)	0.2	7.1×10^4	127.95	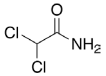
trichloroacetamide (TCAM)	1	1.3×10^4	162.40	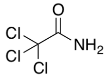
monobromoacetonitrile (MBAN)	0.20	1.0×10^2	119.95	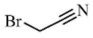
dibromoacetonitrile (DBAN)	0.47	9.6×10^3	198.84	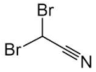
tribromoacetonitrile (TBAN)	2.3	Not available	277.74	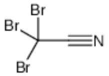
monobromoacetamide (MBAM)	-0.5	1.6×10^5	137.96	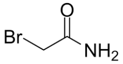
dibromoacetamide (DBAM)	0.8	1.0×10^5	216.86	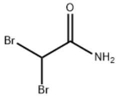
tribromoacetamide (TBAM)	1.5	Not available	295.76	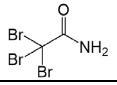

Table S2. Quantum chemical parameters used in this study, and were calculated using Orca electronic structure package.

DBPs	E_{HOMO}	E_{LUMO}	E_{LUMO}-E_{HOMO}	Electro negativity (χ)	Electrophilicity index (ω)	Ionization potential (*IE*)	Electron affinity (*EA*)	Hardness (η)	Softness (σ)
MCAN	-8.987	-1.475	7.512	5.231	1.822	11.530	1.415	7.512	0.133
DCAN	-9.206	-1.854	7.352	5.530	2.080	11.414	1.843	7.352	0.136
TCAN	-9.198	-2.156	7.042	5.677	2.289	11.426	1.991	7.042	0.142
MCAM	-7.523	-0.426	7.097	3.974	1.113	9.759	1.303	7.097	0.141
DCAM	-7.826	-1.247	6.579	4.537	1.564	9.867	1.572	6.579	0.152
TCAM	-8.036	-1.539	6.497	4.788	1.764	9.926	2.030	6.497	0.154
MBAN	-8.400	-1.996	6.404	5.198	2.109	10.955	1.650	6.404	0.156
DBAN	-8.433	-2.560	5.872	5.496	2.572	10.470	1.968	5.872	0.170
TBAN	-8.332	-3.047	5.286	5.689	3.062	10.394	2.288	5.286	0.189
MBAM	-7.475	-0.927	6.548	4.201	1.348	9.600	1.505	6.548	0.153
DBAM	-7.717	-1.792	5.925	4.755	1.908	9.638	1.771	5.925	0.169
TBAM	-7.758	-2.464	5.294	5.111	2.467	9.404	2.263	5.294	0.189

E_{HOMO} (eV) = energy of the highest occupied molecular orbital. E_{LUMO} (eV) = energy of the lowest unoccupied molecular orbital. E_{gap} (eV) = E_{LUMO}-E_{HOMO}. *IP* (eV) = ionization potential. *EA* (eV) = electron affinity. η (hardness, eV) = (IP-EA)/2. *S* (softness, eV^{-1}) = 1/(*IP*-*EA*). χ (electronegativity, eV) = (*IP*+*EA*)/2. ω (electrophilic index, eV) = $\chi^2/2\eta$.

Table S3. Fluence rate with corrections using water factor (WF).

Water matrix	Absorbance	Water factor (WF)	Fluence rate (I_0; Einstein·L^{-1}·s^{-1})	Fluence rate after correction (I_{ave}=WF× I_0; Einstein·L^{-1}·s^{-1})
DI water	0	1	3.14×10^{-6}	3.14×10^{-6}
1 ppm NO_3^-	0.048	0.881		2.77×10^{-6}
2 ppm NO_3^-	0.109	0.756		2.37×10^{-6}
5 ppm NO_3^-	0.273	0.524		1.64×10^{-6}
10 ppm NO_3^-	0.596	0.299		9.39×10^{-7}
1 ppm FA	0.019	0.951		2.98×10^{-6}
2 ppm FA	0.049	0.879		2.76×10^{-6}
5 ppm FA	0.116	0.744		2.33×10^{-6}
10 ppm FA	0.248	0.552		1.73×10^{-6}
Simulated water	0.061	0.881		2.68×10^{-6}
1 mM TBA	0	1	3.14×10^{-6}	3.14×10^{-6}

Table S4. Decay % and rate constant (*k*) for DCAM photodegrdation at 222 nm in the presence of water matrix constitutent.

water matrix constituent	concentration (mg·L^{-1})	$[DCAM]_{decay,\%}$ [a]	time-based rate constant (sec^{-1})	fluence-based rate constant (L·Einstein^{-1})	adjusted fluence-based rate constant (L·Einstein^{-1})[b]
No constituent		92.7	$(2.84 \pm 0.26) \times 10^{-3}$	$(9.04 \pm 0.84) \times 10^{2}$	$(9.04 \pm 0.84) \times 10^{2}$
Nitrate	1	91.1	$(1.36 \pm 0.04) \times 10^{-3}$	$(4.33 \pm 0.13) \times 10^{2}$	$(4.92 \pm 0.15) \times 10^{2}$
	2	90.6	$(1.16 \pm 0.04) \times 10^{-3}$	$(5.23 \pm 0.14) \times 10^{2}$	$(6.91 \pm 0.19) \times 10^{2}$
	5	88.1	$(1.23 \pm 0.05) \times 10^{-3}$	$(4.30 \pm 0.27) \times 10^{2}$	$(8.20 \pm 0.52) \times 10^{2}$
	10	77.7	$(1.23 \pm 0.01) \times 10^{-3}$	$(3.90 \pm 0.39) \times 10^{2}$	$(1.31 \pm 0.13) \times 10^{3}$
Fulvic acids	2	95.3	$(1.73 \pm 0.04) \times 10^{-3}$	$(5.50 \pm 0.14) \times 10^{2}$	$(6.26 \pm 0.16) \times 10^{2}$
	5	94.1	$(1.74 \pm 0.08) \times 10^{-3}$	$(5.54 \pm 0.27) \times 10^{2}$	$(7.45 \pm 0.36) \times 10^{2}$
Simulated water		95.2	$(1.78 \pm 0.05) \times 10^{-3}$	$(5.64 \pm 0.15) \times 10^{2}$	$(6.62 \pm 0.18) \times 10^{2}$

[a] Degree of DBP decay for 30 min.

[b] fluence rate was corrected by water factor in the presence of water matrix constituent(s).

Table S5. Comparison of molar absorption coefficient (ε) and quantum yield (Φ) at 222 nm and 254 nm.

DBPs	ε; 222 nm/254nm	Φ; 222 nm/254nm
MCAN	7.0	2.1
DCAN	7.0	3.4
TCAN	3.5	3.5
MCAM	3.5×10^{1}	1.6
DCAM	3.0×10^{1}	1.4
TCAM	3.0×10^{1}	1.5
MBAN	5.1	4.5
DBAN	2.3	1.3
TBAN	2.4	1.9
MBAM	1.1×10^{1}	1.3
DBAM	7.8	1.2
TBAM	3.3	1.3

Table S6. Rate constants (k) for photolysis of DBPs at 254 nm.

DBP compound	time-based rate constant (k; sec^{-1})	fluence-based rate constant (k)		k 222/254	
		k_V: L·Einstein^{-1}	k_A: cm^2·Einstein^{-1} [a]	$k_{V,222}$ / $k_{V,254}$	$k_{A,222}$ / $k_{A,254}$
MCAN	$(4.33 \pm 0.53) \times 10^{-6}$	(1.94 ± 0.22)	$(5.48 \pm 0.63) \times 10^{2}$	9.6	14.5
DCAN	$(2.19 \pm 0.30) \times 10^{-5}$	(9.82 ± 0.13)	$(2.77 \pm 0.36) \times 10^{3}$	15.5	23.5
TCAN	$(1.25 \pm 0.09) \times 10^{-4}$	$(5.62 \pm 0.38) \times 10^{1}$	$(1.59 \pm 0.11) \times 10^{4}$	8.1	12.3
MCAM	$(1.72 \pm 0.16) \times 10^{-5}$	(7.72 ± 0.69)	$(2.18 \pm 0.20) \times 10^{3}$	36.8	55.8
DCAM	$(7.28 \pm 0.36) \times 10^{-5}$	$(3.26 \pm 0.15) \times 10^{1}$	$(9.21 \pm 0.43) \times 10^{3}$	27.7	42.0
TCAM	$(1.21 \pm 0.04) \times 10^{-4}$	$(5.41 \pm 0.18) \times 10^{1}$	$(1.53 \pm 0.05) \times 10^{4}$	29.1	44.2
MBAN	$(1.55 \pm 0.04) \times 10^{-5}$	(6.96 ± 0.18)	$(1.96 \pm 0.05) \times 10^{3}$	15.0	22.7
DBAN	$(1.37 \pm 0.04) \times 10^{-3}$	$(6.14 \pm 0.18) \times 10^{2}$	$(1.73 \pm 0.05) \times 10^{5}$	2.0	3.0
TBAN	$(2.13 \pm 0.04) \times 10^{-3}$	$(9.56 \pm 0.18) \times 10^{2}$	$(2.70 \pm 0.05) \times 10^{5}$	2.9	4.4
MBAM	$(7.89 \pm 0.04) \times 10^{-5}$	$(3.54 \pm 0.18) \times 10^{1}$	$(9.98 \pm 0.05) \times 10^{3}$	9.4	14.3
DBAM	$(9.34 \pm 0.04) \times 10^{-4}$	$(4.19 \pm 0.18) \times 10^{2}$	$(1.18 \pm 0.05) \times 10^{5}$	6.2	9.4
TBAM	$(2.89 \pm 0.04) \times 10^{-3}$	$(1.30 \pm 0.18) \times 10^{3}$	$(3.66 \pm 0.05) \times 10^{5}$	2.9	4.4

[a] Fluence-based k (in cm^2·Einstein^{-1}) was calculated by dividing the fluence-based k (in L·Einstein^{-1}) with effective path length (l = 3.5 cm).

Table S7. Fluecne required to achieve the removal of half of DBP concentration ($E_{50\%}$; mJ·cm^2).

DBPs	222 nm	254 nm	254 nm/222 nm
MCAN	4.69×10^4	5.97×10^5	12.7
DCAN	5.74×10^3	1.18×10^5	20.1
TCAN	1.92×10^3	2.06×10^4	10.8
MCAM	3.07×10^3	1.50×10^5	48.8
DCAM	9.67×10^2	3.55×10^4	36.7
TCAM	5.53×10^2	2.14×10^4	38.7
MBAN	8.37×10^3	1.66×10^5	19.9
DBAN	7.08×10^2	1.89×10^3	2.7
TBAN	3.14×10^2	1.21×10^3	3.9
MBAM	2.62×10^3	3.27×10^4	12.5
DBAM	3.37×10^2	2.77×10^3	8.2
TBAM	2.33×10^2	8.94×10^2	3.8

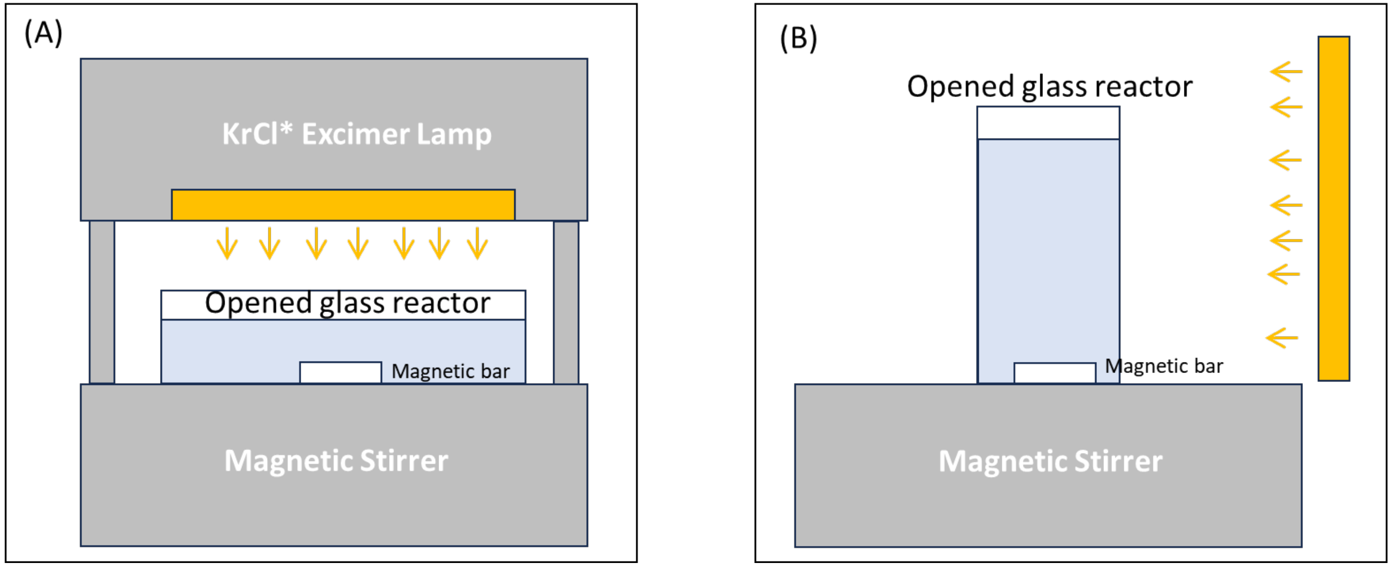

Figure S1. UV set-up for (A) KrCl* Excimer lamp emitting at 222 nm and (B) LPUV lamp emitting at 254 nm.

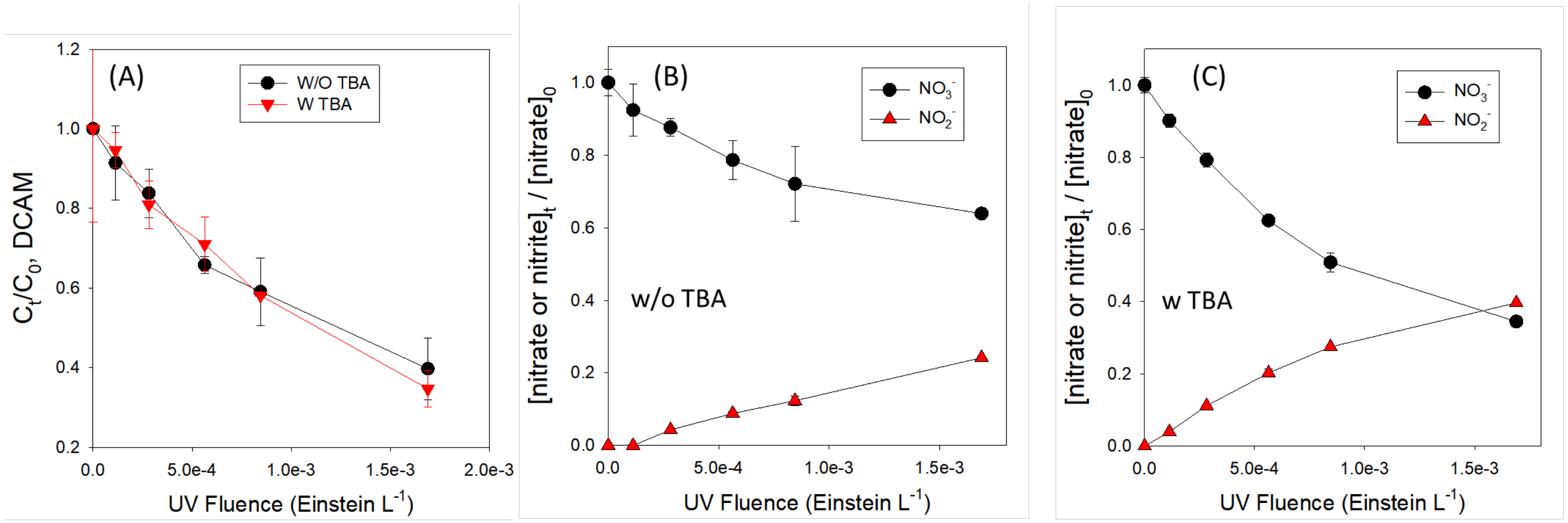

Figure S2. (A) Photolysis of dichloroacetamide (DCAM) at 222 nm with or without TBA in the presence of nitrate. (B and C) Nitrite formation at 222 nm with or without TBA in the presence of nitrate. Reaction conditions: $[DCAM]_0 = 100\ \mu g \cdot L^{-1}$, pH = 7.0, 10 mM phosphate, [TBA] = 0 or 1 mM, $[nitrate]_0 = 10\ mg \cdot L^{-1}$.

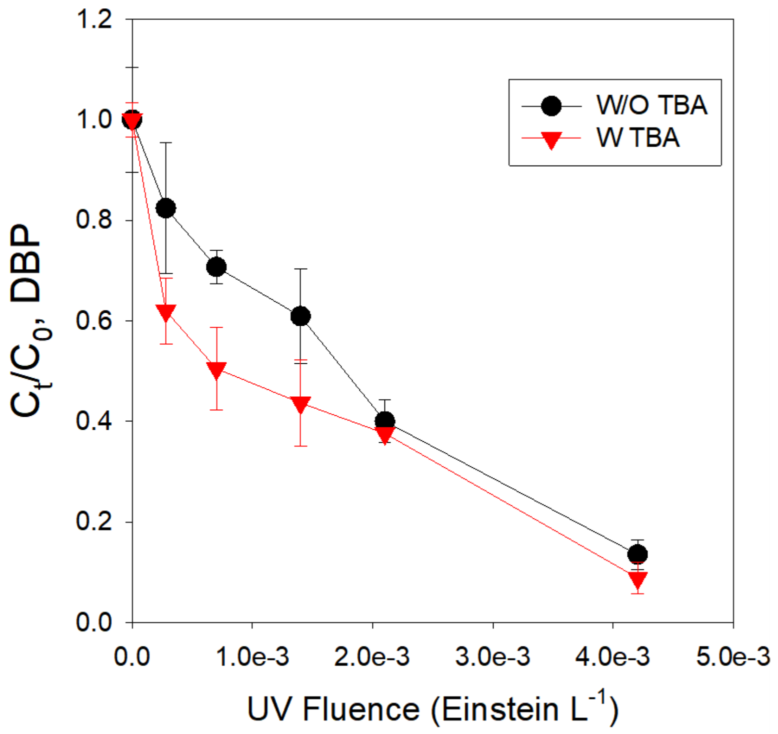

Figure S3. Photolysis of dichloroacetamide (DCAM) at 222 nm with or without TBA in the presence of fulvic acid. Reaction conditions: $[DCAM]_0$ = 100 μg·L^{-1}, pH = 7.0, 10 mM phosphate, [TBA] = 0 or 1 mM, $[\text{fulvic acid}]_0$ = 5 mg·L^{-1}

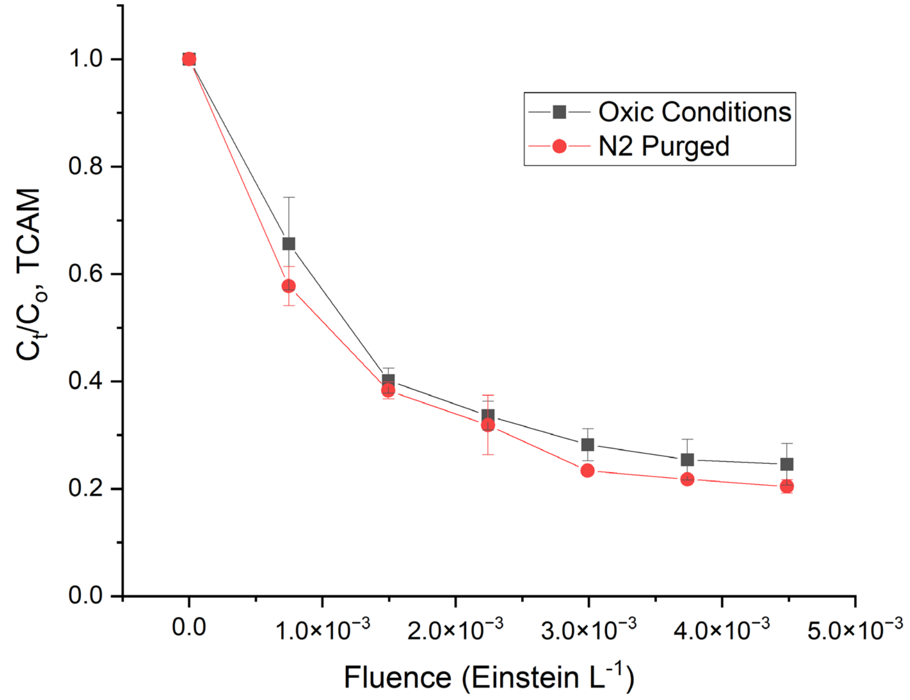

Figure S4. Photolysis of tricloroacetamide (TCAM) at 222 nm with or without being purged with N_2 gas prior to irradiation. Reaction conditions: $[TCAM]_0 = 100$ μg·L^{-1}, pH = 6.7, 10 mM phosphate, $E_o = 2.49\times10^{-6}$ Einstein/L/sec. Error bars display standard deviation from the average values of duplicate experiments.

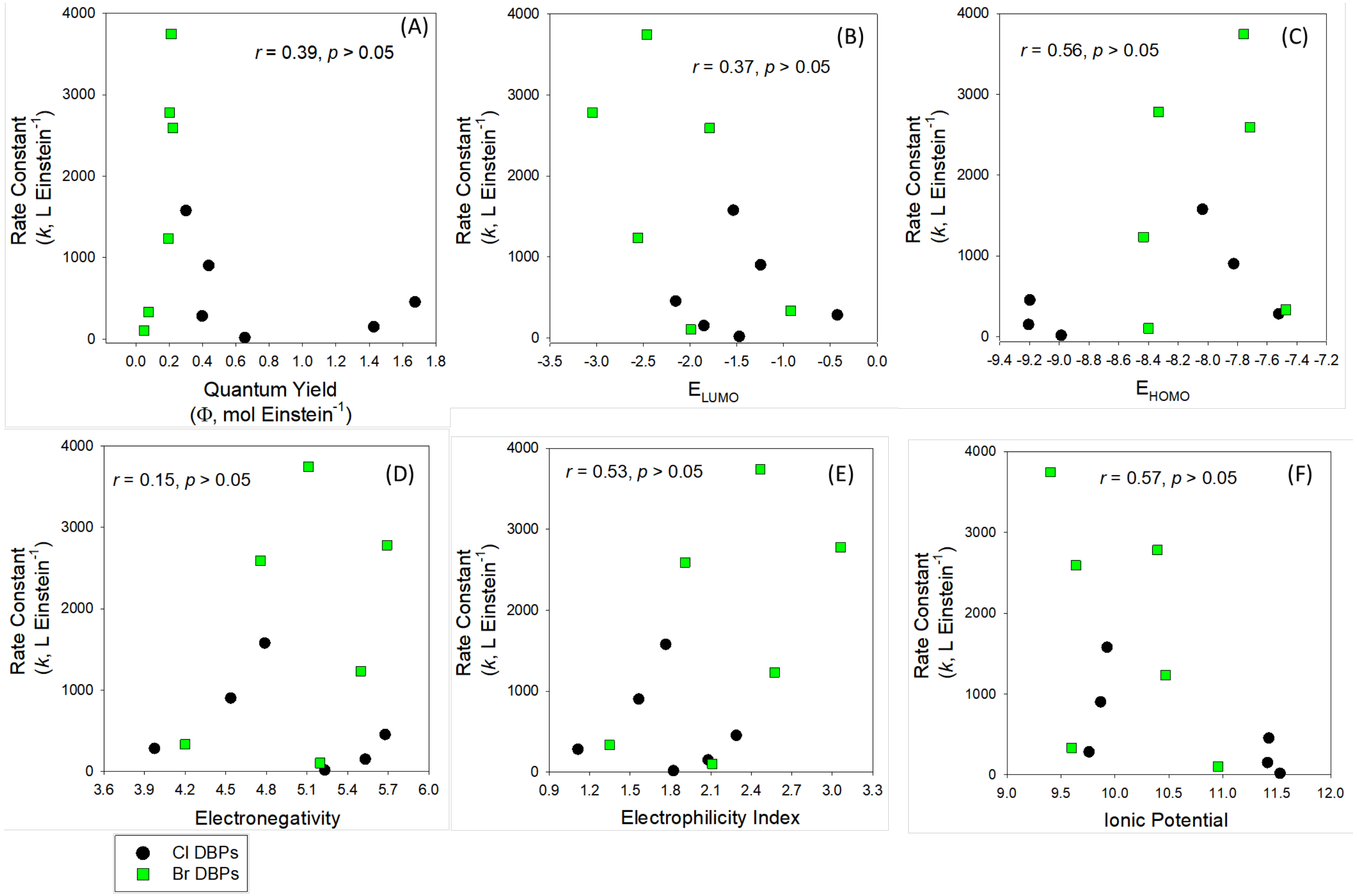

Figure S5. Poor correlation between rate constants (k, L·Einstein^{-1}) for DBP photodegradation at 222 nm with (A) quantum yield (Φ), (B) E_{LOMO}, (C) E_{HOMO}, (D) Electronegativity, (E) Electrophilicity Index, and (F) Ionization Potential.

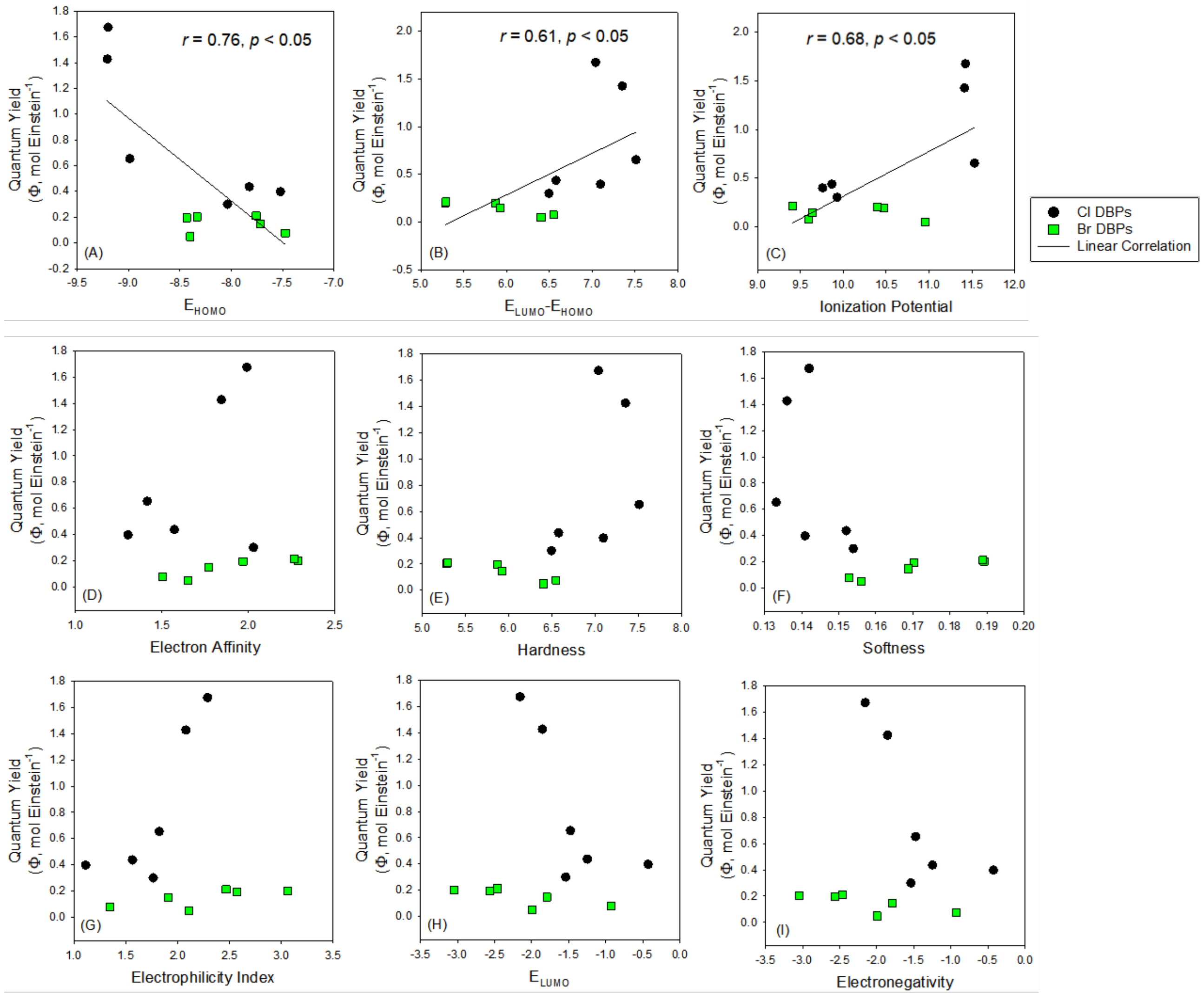

Figure S6. Correlation between quantum yield (mol·Einstein^{-1}) of DBP at 222 nm with (A) E_{HOMO}, (B) E_{LUMO} - E_{HOMO} (C) Ionization Potential, (D) Electron Affinity, (E) Hardness, (F) Softness, (G) Electrophilicity Index, (H) E_{LOMO}, and (I) Electronegativity.

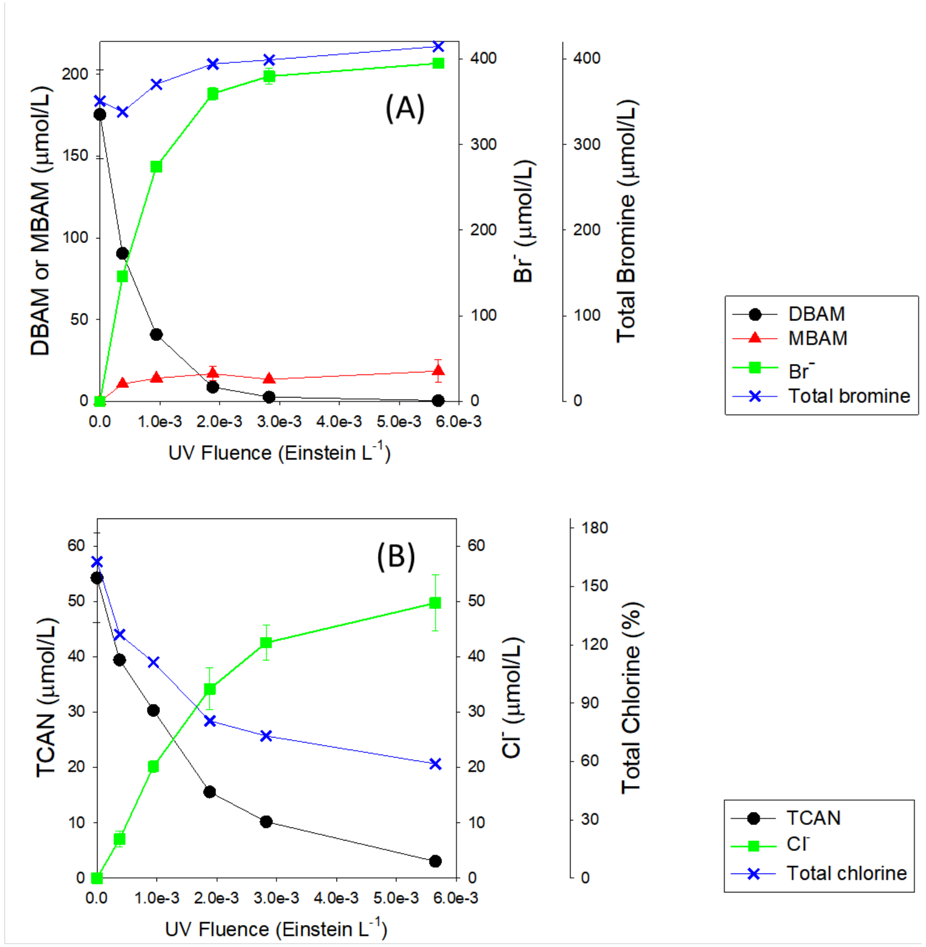

Figure S7. Transformation products of DBAM (A) and TCAN (B) at 222 nm. Reaction condition: $[DBAM]_0$ = 175 μmol·L^{-1}, $[TCAN]_0$ = 54 μmol·L^{-1}, pH = 6.9, 10 mM phosphate. Total bromine and total chlorine represent the sum of bromine or chlorine in the parent DBP, transformation products, and Br^- or Cl^- generated during the reaction.

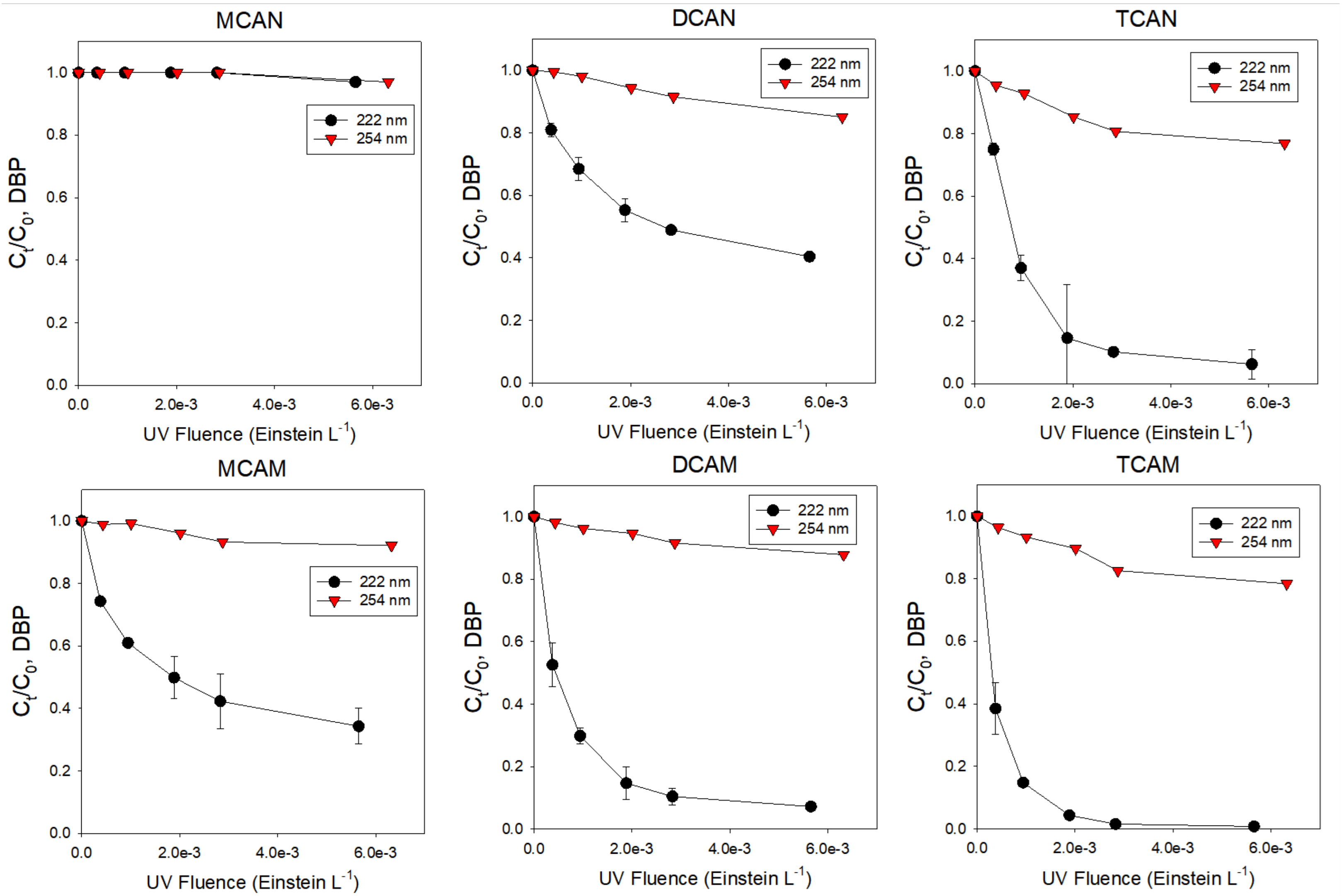

Figure S8. Comparison of photolysis of chloro-DBPs at different irradiation wavelengths (222-nm and 254-nm) but on similar fluence bases. Reaction condition: $[DBP]_0$ = 100 μg·L^{-1}, pH = 6.9, 10 mM phosphate.

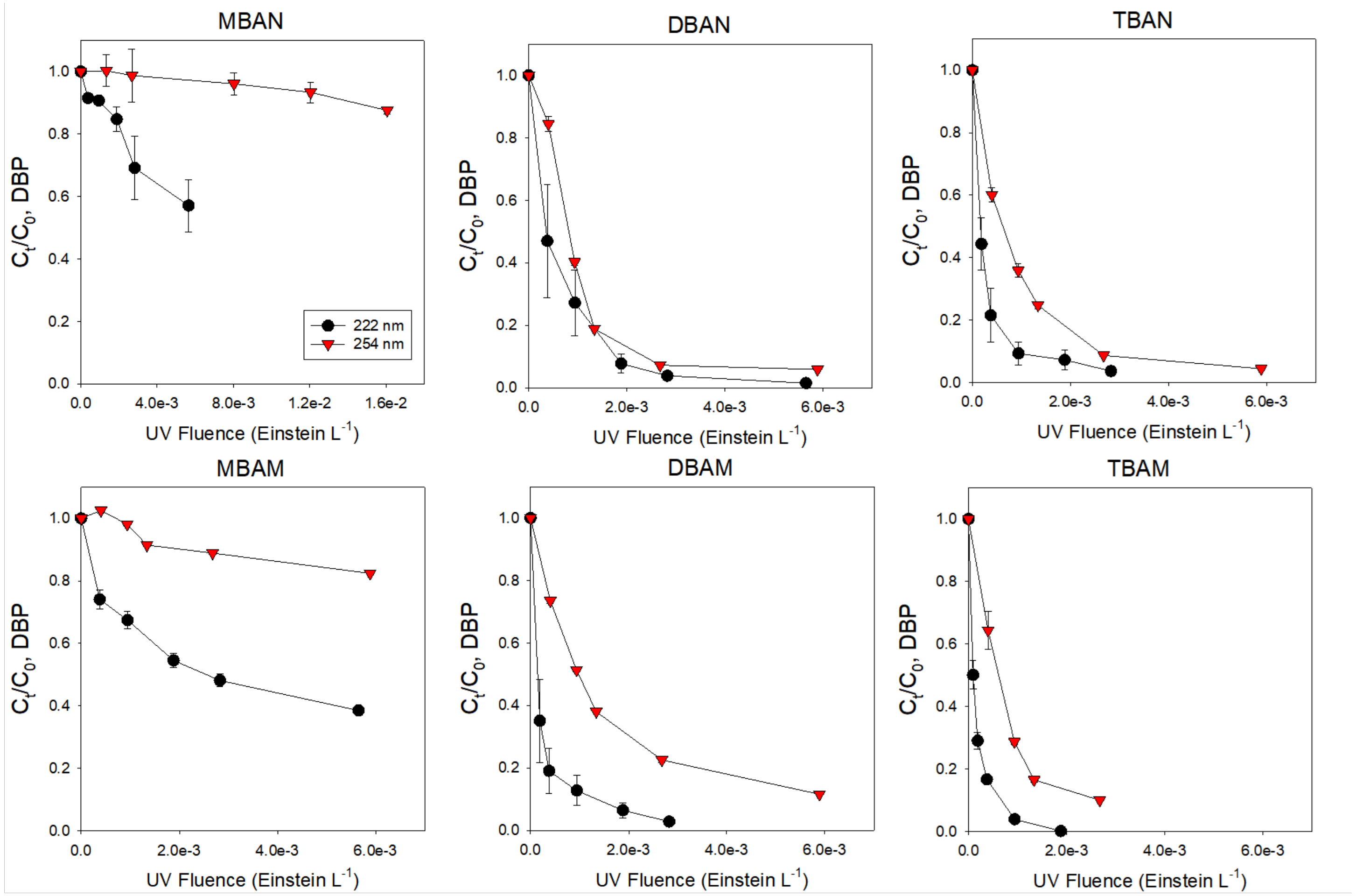

Figure S9. Comparison of photolysis of bromo-DBPs at different irradiation wavelengths (222-nm and 254-nm) but on similar fluence bases. Reaction condition: $[DBP]_0$ = μg·L^{-1}, pH = 6.9, 10 mM phosphate.

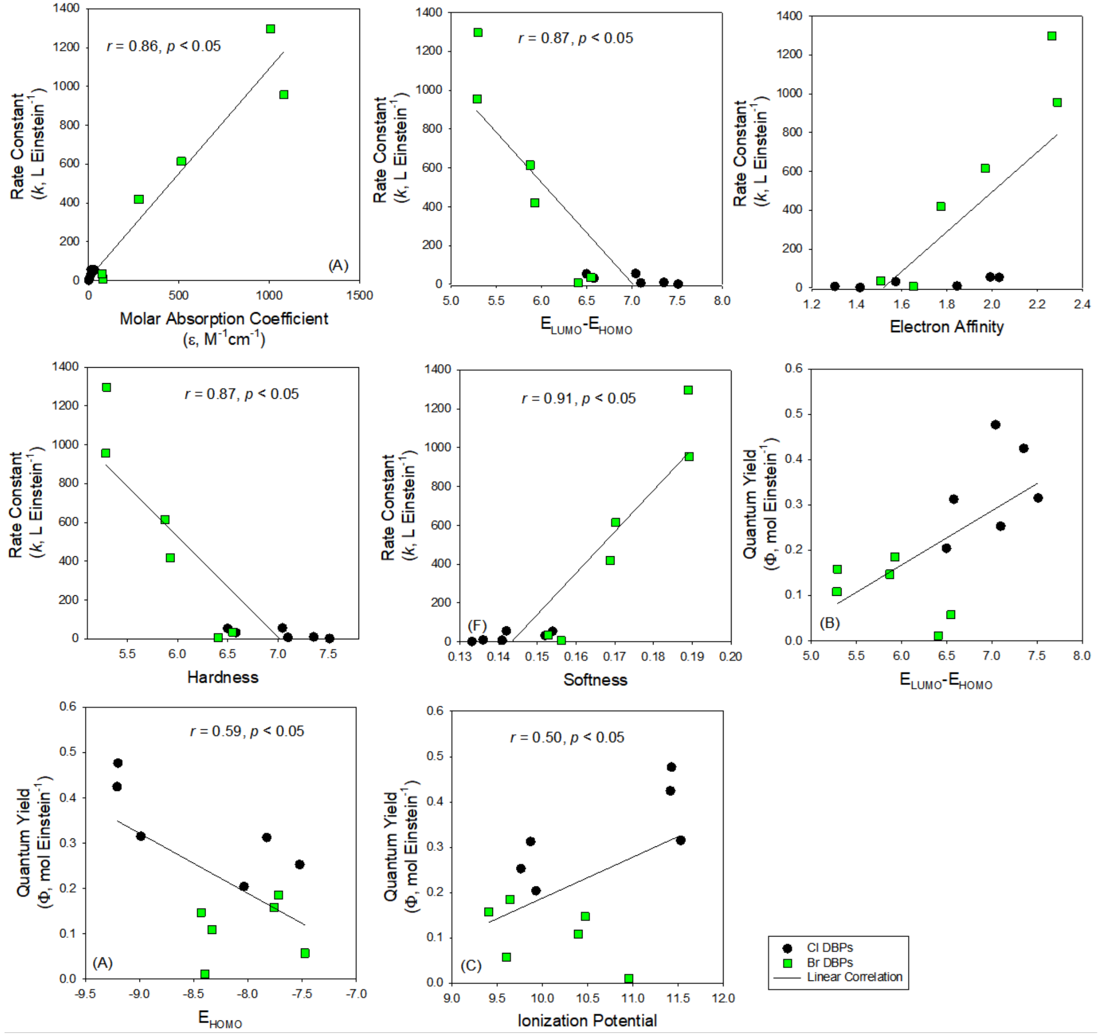

Figure S10. Correlation between rate constants (k, L·Einstein^{-1}) for DBP photodegradation or quantum yield (mol·Einstein^{-1}) of DBP at 254 nm with molar absorption coefficient and quantum chemical parameters.
